# Supplementary material for: The Evolution and Characterization of the RNA Interference Pathways in Lophotrochozoa
Source: Genome Biol Evol. 2024 May 7;16(5):evae098. doi: 10.1093/gbe/evae098 (PMC11114477; doi:10.1093/gbe/evae098)
Supplement: evae098_Supplementary_Data [file evae098_supplementary_data.zip › Supplementary_materials.zip!/selected_miRNAs/NoGe_selected_miRNAs/mirdeep_pdf_files/NMRB01000014.1_Notospermus_geniculatus_strain_Ushimado_scaffold14,_whole_genome_shotgun_sequence_617.pdf]

Provisional ID	:	NMRB01000014.1_Notospermus_geniculatus_strain_Ushimado_scaffold14,_whole_genome_shotgun_sequence_617
Score total	:	977762.4
Score for star read(s)	:	3.9
Score for read counts	:	977755.5
Score for mfe	:	0
Score for randfold	:	0
Score for cons. seed	:	3
Total read count	:	1917837
Mature read count	:	1917797
Loop read count	:	0
Star read count	:	40

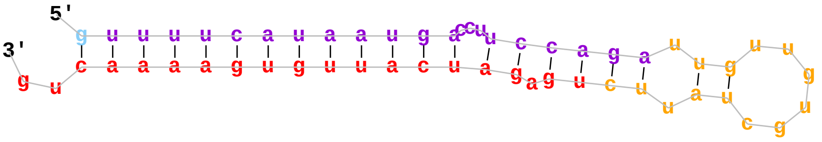

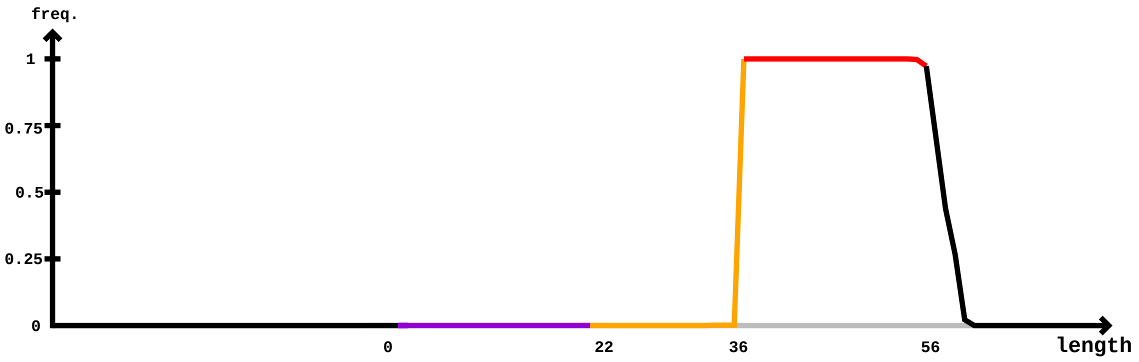

Star Mature

```
5'- uguuggcuguaaugaugaccuucagacgaaacugguuuucauaaugaccuuccagauuguugugcuauucugagaucauugugaaaacugauuccguugcccccaauc   -3'   obs
    uguuggcuguaaugaugaccuucagacgaaacugguuuucauaaugaccuuccagauuguugugcuauucugagaucauugugaaaacugauuccguugcccccaauc         exp
    .(((((..((((((........(((......)))((((((((((((...((((((.((......)).)))).)))))))))))))))......))))))..))))).    reads   mm   sample
    ...............................cugguuuucauaaugaccuuccag.....................................................    2       0    Mm3
    ...............................cugguuuucauaaugaccuuccaga....................................................    3       0    Mm3
    ...................................uuuucauaaugaccuuAcaga....................................................    1       1    Mm3
    ...................................uuuucauaaugaccuuccaga....................................................    1       0    Mm3
    ........................................................uuguugugcuauucugagaucauugug.........................    1       0    Mm3
    ...................................................................uucugagaucauugugaaaacG...................    1       1    Mm3
    ...................................................................uucugagaucauugugaaaacu...................    63      0    Mm3
    ...................................................................uucugagaucGuugugaaaacu...................    1       1    Mm3
    ...................................................................uucugagaucauugugaaaacuU..................    1       1    Mm3
    ....................................................................ucugagaucauugugaaaacug..................    1       0    Mm3
    .....................................................................cugagaucauugugaaaacugauu...............    1       0    Mm3
    ......................................................................ugagaucauugugaaaaU....................    6       1    Mm3
    ......................................................................ugagaucauugugaaaaG....................    12      1    Mm3
    ......................................................................ugagaucauugugaaaac....................    51      0    Mm3
    ......................................................................ugagaucauugugaaaaA....................    25      1    Mm3
    ......................................................................ugagaucauugugaaaUcu...................    5       1    Mm3
    ......................................................................Cgagaucauugugaaaacu...................    1       1    Mm3
    ......................................................................ugGgaucauugugaaaacu...................    1       1    Mm3
    ......................................................................ugagaucGuugugaaaacu...................    4       1    Mm3
    ......................................................................ugagaucauugugaaaaAu...................    16      1    Mm3
    ......................................................................ugagaucauugugaaaacA...................    1       1    Mm3
    ......................................................................ugagaucauuCugaaaacu...................    1       1    Mm3
    ......................................................................ugagaucauugugaaaaUu...................    7       1    Mm3
    ......................................................................Ngagaucauugugaaaacu...................    1       1    Mm3
    ......................................................................ugagaucauugugaaaGcu...................    1       1    Mm3
    ......................................................................ugUgaucauugugaaaacu...................    2       1    Mm3
    ......................................................................ugagaucauugugGaaacu...................    2       1    Mm3
    ......................................................................ugagGucauugugaaaacu...................    1       1    Mm3
    ......................................................................ugagaucauugugaaaCcu...................    3       1    Mm3
    ......................................................................ugagaucauugugaaaacG...................    2       1    Mm3
    ......................................................................ugagaucauugugaaGacu...................    3       1    Mm3
    ......................................................................ugagaucauugugaaaaGu...................    5       1    Mm3
    ......................................................................ugagaucauugugaGaacu...................    1       1    Mm3
```

Star Mature

```
uguuggcuguaaugaugaccuucagacgaaacugguuuucauaaugaccuuccagauuguugugcuauucugagaucauugugaaaacugauuccguugcccccaauc
......................................................................ugagaucauugugaaaacu...................   1295   0   Mm3
......................................................................ugagaucauuUugaaaacug..................   14     1   Mm3
......................................................................Agagaucauugugaaaacug..................   2      1   Mm3
......................................................................ugaAaucauugugaaaacug..................   1      1   Mm3
......................................................................ugagaucauugAgaaaacug..................   2      1   Mm3
......................................................................ugagaucauugugaaaacuU..................   23     1   Mm3
......................................................................uNagaucauugugaaaacug..................   1      1   Mm3
......................................................................ugagaucauuAugaaaacug..................   16     1   Mm3
......................................................................ugagaucauugugaaaacGg..................   20     1   Mm3
......................................................................ugagaucauugugaaaacug..................   9259   0   Mm3
......................................................................ugUgaucauugugaaaacug..................   1      1   Mm3
......................................................................ugagaGcauugugaaaacug..................   1      1   Mm3
......................................................................Ggagaucauugugaaaacug..................   2      1   Mm3
......................................................................ugagaucauugugaaaaAug..................   76     1   Mm3
......................................................................ugagaucauugugaaaacuC..................   1      1   Mm3
......................................................................ugagaucauugugaaaacuA..................   52     1   Mm3
......................................................................uCagaucauugugaaaacug..................   1      1   Mm3
......................................................................ugagaucauugugGaaacug..................   1      1   Mm3
......................................................................Cgagaucauugugaaaacug..................   7      1   Mm3
......................................................................uAagaucauugugaaaacug..................   7      1   Mm3
......................................................................ugagaucauCgugaaaacug..................   3      1   Mm3
......................................................................ugagUucauugugaaaacug..................   1      1   Mm3
......................................................................ugagaucauugugaaaUcug..................   12     1   Mm3
......................................................................Ngagaucauugugaaaacug..................   5      1   Mm3
......................................................................ugagaucauugugaaaacAg..................   1      1   Mm3
......................................................................ugagaucauugugaaaCcug..................   19     1   Mm3
......................................................................ugagaucauugugUaaacug..................   2      1   Mm3
......................................................................ugagaucauugugaaaaUug..................   58     1   Mm3
......................................................................ugagaucauuCugaaaacug..................   14     1   Mm3
......................................................................ugagaucauugugaaaacCg..................   1      1   Mm3
......................................................................ugagaAcauugugaaaacug..................   2      1   Mm3
......................................................................ugagaucauugugaaUacug..................   2      1   Mm3
......................................................................ugGgaucauugugaaaacug..................   3      1   Mm3
......................................................................ugagCucauugugaaaacug..................   5      1   Mm3
......................................................................ugagauGauugugaaaacug..................   1      1   Mm3
......................................................................ugagauUauugugaaaacug..................   1      1   Mm3
......................................................................ugagaucauugugaaaaGug..................   36     1   Mm3
......................................................................ugagaucauugugaUaacug..................   2      1   Mm3
......................................................................ugagaucGuugugaaaacug..................   4      1   Mm3
......................................................................ugagaucauugugaaGacug..................   4      1   Mm3
......................................................................ugagaucauuguAaaaacug..................   2      1   Mm3
......................................................................ugagaucauugugaaaGcug..................   15     1   Mm3
......................................................................ugCgaucauugugaaaacug..................   1      1   Mm3
......................................................................uUagaucauugugaaaacug..................   1      1   Mm3
......................................................................ugagaucaAugugaaaacug..................   1      1   Mm3
......................................................................ugagaucauugugaaaacugU.................   282    1   Mm3
......................................................................ugagaucauugugaaaaUuga.................   87     1   Mm3
......................................................................ugagaucauugugaaUacuga.................   1      1   Mm3
......................................................................ugagaucauuAugaaaacuga.................   24     1   Mm3
......................................................................ugagaucauCgugaaaacuga.................   2      1   Mm3
......................................................................ugagaucauugugaaaCcuga.................   22     1   Mm3
......................................................................ugCgaucauugugaaaacuga.................   1      1   Mm3
......................................................................ugagaucauuguAaaaacuga.................   3      1   Mm3
......................................................................ugagauUauugugaaaacuga.................   5      1   Mm3
......................................................................ugagCucauugugaaaacuga.................   3      1   Mm3
......................................................................ugagaucUuugugaaaacuga.................   9      1   Mm3
......................................................................ugagaCcauugugaaaacuga.................   2      1   Mm3
......................................................................ugagaucauugugGaaacuga.................   3      1   Mm3
......................................................................ugagaucauugugaaaacuga.................   15493  0   Mm3
......................................................................ugagaucauugugaUaacuga.................   1      1   Mm3
......................................................................ugagaucauugugaaaaAuga.................   121    1   Mm3
......................................................................ugagaucauugugaaaUcuga.................   28     1   Mm3
......................................................................ugagaucauugugaaaacCga.................   2      1   Mm3
......................................................................ugagaucauugugaaaacugC.................   8      1   Mm3
......................................................................uUagaucauugugaaaacuga.................   2      1   Mm3
......................................................................ugagaucauugugaGaacuga.................   3      1   Mm3
......................................................................Cgagaucauugugaaaacuga.................   5      1   Mm3
......................................................................ugagaucauuCugaaaacuga.................   14     1   Mm3
......................................................................ugagaucGuugugaaaacuga.................   5      1   Mm3
......................................................................ugagaucauuUugaaaacuga.................   13     1   Mm3
```

```
                                           Star                      Mature
uguuggcuguaaugaugaccuucagacgaaacugguuuucauaaugaccuuccagauuguugugcuauucugagaucauugugaaaacugauuccguugcccccaauc
......................................................................ugagaucauugugaaGacuga.................	6	1	Mm3
......................................................................ugagaucauugCgaaaacuga.................	1	1	Mm3
......................................................................ugagaucauugugaaaacuAa.................	1	1	Mm3
......................................................................ugagaucauugugaaaacuCa.................	2	1	Mm3
......................................................................ugUgaucauugugaaaacuga.................	3	1	Mm3
......................................................................ugagaucauugugUaaacuga.................	1	1	Mm3
......................................................................Ngagaucauugugaaaacuga.................	5	1	Mm3
......................................................................ugagaNcauugugaaaacuga.................	1	1	Mm3
......................................................................ugagaucauugugaaaacugG.................	217	1	Mm3
......................................................................uAagaucauugugaaaacuga.................	5	1	Mm3
......................................................................ugagaucaCugugaaaacuga.................	2	1	Mm3
......................................................................ugagaucauugugaaaaGuga.................	65	1	Mm3
......................................................................ugGgaucauugugaaaacuga.................	2	1	Mm3
......................................................................ugagaAcauugugaaaacuga.................	1	1	Mm3
......................................................................ugNgaucauugugaaaacuga.................	1	1	Mm3
......................................................................ugagaucauugugaaaacuUa.................	2	1	Mm3
......................................................................ugagaucauuguCaaaacuga.................	3	1	Mm3
......................................................................ugagaucauAgugaaaacuga.................	2	1	Mm3
......................................................................ugagaucauugugaaaGcuga.................	18	1	Mm3
......................................................................ugaUaucauugugaaaacuga.................	1	1	Mm3
......................................................................ugagGucauugugaaaacuga.................	1	1	Mm3
......................................................................ugagaucauugugaaaacugUu................	9	1	Mm3
......................................................................ugagauUauugugaaaacugau................	5	1	Mm3
......................................................................ugagaucauugugaGaacugau................	4	1	Mm3
......................................................................ugagaucauugugaaCacugau................	1	1	Mm3
......................................................................ugagaucauugugaaaacAgau................	1	1	Mm3
......................................................................ugagaucauugCgaaaacugau................	1	1	Mm3
......................................................................ugagaNcauugugaaaacugau................	1	1	Mm3
......................................................................ugagaucGuugugaaaacugau................	5	1	Mm3
......................................................................ugagaucauAgugaaaacugau................	2	1	Mm3
......................................................................ugagaucauugugaaaacugaG................	1	1	Mm3
......................................................................ugagaucauugugaaaacuCau................	2	1	Mm3
......................................................................ugagaucauuAugaaaacugau................	27	1	Mm3
......................................................................Agagaucauugugaaaacugau................	2	1	Mm3
......................................................................ugagaucUuugugaaaacugau................	3	1	Mm3
......................................................................ugagaucauugugUaaacugau................	1	1	Mm3
......................................................................ugagaucauugugCaaacugau................	3	1	Mm3
......................................................................ugCgaucauugugaaaacugau................	2	1	Mm3
......................................................................ugagaucauugugaaaacuAau................	1	1	Mm3
......................................................................ugagaucauugugaaaacugau................	15605	0	Mm3
......................................................................ugagaucauugAgaaaacugau................	1	1	Mm3
......................................................................ugagaucauugugaaaacugaC................	4	1	Mm3
......................................................................ugagGucauugugaaaacugau................	1	1	Mm3
......................................................................ugagaucauugugaaaacugaA................	115	1	Mm3
......................................................................ugUgaucauugugaaaacugau................	6	1	Mm3
......................................................................ugagaucauugugaaaaGugau................	70	1	Mm3
......................................................................ugagaucauugugaaUacugau................	1	1	Mm3
......................................................................ugagaucauugugaaaacugGu................	3	1	Mm3
......................................................................ugGgaucauugugaaaacugau................	4	1	Mm3
......................................................................uNagaucauugugaaaacugau................	1	1	Mm3
......................................................................ugagCucauugugaaaacugau................	6	1	Mm3
......................................................................ugagaucauugugGaaacugau................	2	1	Mm3
......................................................................ugagaucauuguAaaaacugau................	1	1	Mm3
......................................................................ugagaAcauugugaaaacugau................	1	1	Mm3
......................................................................ugagaucauugugaaaCcugau................	22	1	Mm3
......................................................................ugagaucauugugaaaacugCu................	2	1	Mm3
......................................................................ugagaucauugugaaaGcugau................	24	1	Mm3
......................................................................ugagaucauGgugaaaacugau................	1	1	Mm3
......................................................................ugagaucaGugugaaaacugau................	1	1	Mm3
......................................................................ugagauNauugugaaaacugau................	1	1	Mm3
......................................................................ugagaucauugugaaaaAugau................	129	1	Mm3
......................................................................ugagaucauuCugaaaacugau................	14	1	Mm3
......................................................................Ngagaucauugugaaaacugau................	9	1	Mm3
......................................................................ugagaucauugugaaaaUugau................	71	1	Mm3
......................................................................ugagaGcauugugaaaacugau................	1	1	Mm3
......................................................................ugagaucauuguCaaaacugau................	1	1	Mm3
......................................................................ugagaucauugugaaGacugau................	2	1	Mm3
......................................................................ugagaucCuugugaaaacugau................	2	1	Mm3
......................................................................ugagaucauuguUaaaacugau................	1	1	Mm3
......................................................................ugagaucauugugaaaacuUau................	3	1	Mm3
```

Star **Mature**

uguuggcuguaaugaugaccuucagacgaaacugguuuucauaaugaccuuccagauuguugugcuauucugagaucauugugaaaacugauuccguugcccccaauc			
....ugagaucauugugaaaacCgau................	3	1	Mm3
....uAagaucauugugaaaacugau................	3	1	Mm3
....ugaAaucauugugaaaacugau................	1	1	Mm3
....Cgagaucauugugaaaacugau................	11	1	Mm3
....ugagaucauuUugaaaacugau................	12	1	Mm3
....ugagaucauugugaaaUcugau................	35	1	Mm3
....Ggagaucauugugaaaacugauu...............	2	1	Mm3
....ugagaucauugugaaaacCgauu...............	4	1	Mm3
....Cgagaucauugugaaaacugauu...............	5	1	Mm3
....ugagaucauugugaGaacugauu...............	2	1	Mm3
....ugagaucauuAugaaaacugauu...............	40	1	Mm3
....ugagaucauuguUaaaacugauu...............	2	1	Mm3
....ugGgaucauugugaaaacugauu...............	10	1	Mm3
....ugagaucauugugaUaacugauu...............	1	1	Mm3
....ugagUucauugugaaaacugauu...............	2	1	Mm3
....ugagaucCuugugaaaacugauu...............	2	1	Mm3
....ugagGucauugugaaaacugauu...............	4	1	Mm3
....ugCgaucauugugaaaacugauu...............	2	1	Mm3
....Ngagaucauugugaaaacugauu...............	9	1	Mm3
....ugagaucauugugaaaacuAauu...............	1	1	Mm3
....ugagaucauugugaaaaGugauu...............	84	1	Mm3
....ugagCucauugugaaaacugauu...............	8	1	Mm3
....ugagaucauuguAaaaacugauu...............	3	1	Mm3
....ugagaucauugugaaGacugauu...............	5	1	Mm3
....ugagaucGuugugaaaacugauu...............	11	1	Mm3
....ugagaucauugugaaaaAugauu...............	149	1	Mm3
....ugagaucauuUugaaaacugauu...............	16	1	Mm3
....ugagaucauugugGaaacugauu...............	3	1	Mm3
....ugagaucauugugaaaacugaCu...............	5	1	Mm3
....ugaAaucauugugaaaacugauu...............	1	1	Mm3
....ugagaucauugugaaaacugauu...............	18864	0	Mm3
....uUagaucauugugaaaacugauu...............	3	1	Mm3
....ugagaucauugugaaaacAgauu...............	2	1	Mm3
....ugagaucauugGgaaaacugauu...............	1	1	Mm3
....ugagaucauugCgaaaacugauu...............	5	1	Mm3
....ugagauNauugugaaaacugauu...............	2	1	Mm3
....Agagaucauugugaaaacugauu...............	2	1	Mm3
....ugagaucauugugaaaacugaGu...............	1	1	Mm3
....ugagaucaAugugaaaacugauu...............	1	1	Mm3
....ugagaucauugugaaaacugCuu...............	6	1	Mm3
....ugagaucauGgugaaaacugauu...............	1	1	Mm3
....ugagaucauugugaaaCcugauu...............	41	1	Mm3
....ugagaucauugugaaaacugauA...............	158	1	Mm3
....ugaUaucauugugaaaacugauu...............	1	1	Mm3
....uAagaucauugugaaaacugauu...............	5	1	Mm3
....ugagaucauuCugaaaacugauu...............	16	1	Mm3
....ugagaucauugugaaaacNgauu...............	1	1	Mm3
....ugagaucauugugUaaacugauu...............	4	1	Mm3
....ugagaucauugugaaaGcugauu...............	29	1	Mm3
....ugagaucauugugaaaacugauC...............	6	1	Mm3
....ugagaucaGugugaaaacugauu...............	1	1	Mm3
....ugagaucauugugaaaacuUauu...............	2	1	Mm3
....ugagaucauAgugaaaacugauu...............	1	1	Mm3
....ugUgaucauugugaaaacugauu...............	5	1	Mm3
....ugagaucauugugaaaacugUuu...............	4	1	Mm3
....ugagaucauugugaaaaUugauu...............	93	1	Mm3
....ugagaucaCugugaaaacugauu...............	4	1	Mm3
....ugagaAcauugugaaaacugauu...............	1	1	Mm3
....ugagaucauugugaaaacugauG...............	12	1	Mm3
....ugagaucauugugaaaUcugauu...............	43	1	Mm3
....ugagaucauuguCaaaacugauu...............	1	1	Mm3
....ugagaucauugugaaaacugaAu...............	5	1	Mm3
....ugagauUauugugaaaacugauu...............	6	1	Mm3
....ugagaNcauugugaaaacugauu...............	4	1	Mm3
....ugagaucauugugaaUacugauu...............	2	1	Mm3
....ugagaucauugugaaaCcugauuc..............	1	1	Mm3
....ugagaucauugugaaaacugauuA..............	62	1	Mm3
....ugagaucauugugaaaacugauuU..............	1815	1	Mm3
....ugagaucauugugaaaaAugauuc..............	1	1	Mm3
....ugagaucauugugaaaacugauuc..............	2	0	Mm3

```
                                    Star                                Mature
uguuggcuguaaugaugaccuucagacgaaacugguuuucauaaugaccuuccagauuguugugcuauucugagaucauugugaaaacugauuccguugcccccaauc
......................................................................ugagaucauugugaaaacugauuG..............   1      1      Mm3
.......................................................................gagaucauugugaaaacu...................   1      0      Mm3
.......................................................................gagaucauugugaaaacug..................   8      0      Mm3
.......................................................................gagaucauugCgaaaacuga.................   1      1      Mm3
.......................................................................gagaucauugugaaaacuga.................   1      0      Mm3
.......................................................................gagaucauugugaaaacugau................   1      0      Mm3
.......................................................................gagaucauugugaaaacugauu...............   3      0      Mm3
.......................................................................gagaAcauugugaaaacugauu...............   1      1      Mm3

...............................cugguuuucauaaugaccuuccaga....................................................   2      0      Fm3
...................................uuuucauaaugaccuuAcag.....................................................   1      1      Fm3
...................................uuuucauaaugaccuuccaga....................................................   3      0      Fm3
..................................................................auucugagaucauugugaaaac....................   1      0      Fm3
...................................................................uucugagaucauugugaaa.......................   10     0      Fm3
...................................................................uucugagaucauugugaaaac....................   2      0      Fm3
...................................................................Aucugagaucauugugaaaacu...................   1      1      Fm3
...................................................................uucugagaucauugugaaaGcu...................   1      1      Fm3
...................................................................uucugagaucauugugUaaacu...................   2      1      Fm3
...................................................................uAcugagaucauugugaaaacu...................   1      1      Fm3
...................................................................uucugagaucauuAugaaaacu...................   1      1      Fm3
...................................................................uucugagaucauugugaGaacu...................   1      1      Fm3
...................................................................uucugagaucauugugCaaacu...................   1      1      Fm3
...................................................................uucugagaucauugugaaaacu...................   226    0      Fm3
...................................................................uucugagaucauugugaaaacuC..................   1      1      Fm3
...................................................................uucugagaucauugugaaaacug..................   3      0      Fm3
...................................................................uucugagaucauugugaaaacuA..................   1      1      Fm3
....................................................................ucugagaucauugugaaaacu...................   7      0      Fm3
....................................................................ucugagaucauugugaaaacug..................   4      0      Fm3
.....................................................................cugagaucauugugaaaacug..................   2      0      Fm3
.....................................................................cugagaucauugugaaaacugau................   2      0      Fm3
.....................................................................cugagaucauugugaaaacugauu...............   2      0      Fm3
......................................................................ugagaucauuUugaaaac....................   2      1      Fm3
......................................................................ugagaucauugugaaaCc....................   3      1      Fm3
......................................................................ugagaNcauugugaaaac....................   1      1      Fm3
......................................................................ugaAaucauugugaaaac....................   1      1      Fm3
......................................................................ugagaucauugugaaaaU....................   214    1      Fm3
......................................................................ugagaucauuCugaaaac....................   3      1      Fm3
......................................................................Agagaucauugugaaaac....................   1      1      Fm3
......................................................................ugagCucauugugaaaac....................   1      1      Fm3
......................................................................Cgagaucauugugaaaac....................   1      1      Fm3
......................................................................ugagaucauugugaaaaG....................   564    1      Fm3
......................................................................ugagaucauugugaGaac....................   1      1      Fm3
......................................................................uAagaucauugugaaaac....................   1      1      Fm3
......................................................................ugagauUauugugaaaac....................   1      1      Fm3
......................................................................ugagaucauugugaaaac....................   1347   0      Fm3
......................................................................ugagaucauugugaaaUc....................   3      1      Fm3
......................................................................ugagaucauugugaaaaA....................   606    1      Fm3
......................................................................uUagaucauugugaaaac....................   1      1      Fm3
......................................................................ugUgaucauugugaaaac....................   1      1      Fm3
......................................................................Ngagaucauugugaaaac....................   1      1      Fm3
......................................................................ugagaucGuugugaaaac....................   1      1      Fm3
......................................................................ugagaucauugugaaaGc....................   4      1      Fm3
......................................................................ugagauGauugugaaaacu...................   1      1      Fm3
......................................................................ugagaCcauugugaaaacu...................   1      1      Fm3
......................................................................ugUgaucauugugaaaacu...................   2      1      Fm3
......................................................................ugagaucauugugaaaacA...................   30     1      Fm3
......................................................................ugagaucauugugaaaCcu...................   30     1      Fm3
......................................................................ugagaucaGugugaaaacu...................   3      1      Fm3
......................................................................ugGgaucauugugaaaacu...................   6      1      Fm3
......................................................................ugagaucauugugGaaacu...................   2      1      Fm3
......................................................................ugagaucauCgugaaaacu...................   4      1      Fm3
......................................................................ugCgaucauugugaaaacu...................   4      1      Fm3
......................................................................ugagaucauugugaaaUcu...................   40     1      Fm3
......................................................................Ngagaucauugugaaaacu...................   15     1      Fm3
......................................................................ugaCaucauugugaaaacu...................   1      1      Fm3
......................................................................ugagaucauugugUaaacu...................   1      1      Fm3
......................................................................ugagaucauugCgaaaacu...................   12     1      Fm3
......................................................................ugagaucauuCugaaaacu...................   22     1      Fm3
......................................................................ugaUaucauugugaaaacu...................   3      1      Fm3
......................................................................ugagaucauugugaGaacu...................   4      1      Fm3
```

```
                                          Star                                Mature
uguuggcuguaaugaugaccuucagacgaaacugguuuucauaaugaccuuccagauuguugugcuauucugagaucauugugaaaacugauuccguugcccccaauc
......................................................................Cgagaucauugugaaaacu...................   2        1    Fm3
......................................................................ugagaAcauugugaaaacu...................   6        1    Fm3
......................................................................ugagaucauugugaaaaUu...................   168      1    Fm3
......................................................................ugagCucauugugaaaacu...................   5        1    Fm3
......................................................................ugagauNauugugaaaacu...................   2        1    Fm3
......................................................................ugagaucauugugaaGacu...................   2        1    Fm3
......................................................................uUagaucauugugaaaacu...................   10       1    Fm3
......................................................................uNagaucauugugaaaacu...................   2        1    Fm3
......................................................................ugagGucauugugaaaacu...................   2        1    Fm3
......................................................................Agagaucauugugaaaacu...................   2        1    Fm3
......................................................................ugagaucauugugaaUacu...................   1        1    Fm3
......................................................................ugagaucUuugugaaaacu...................   5        1    Fm3
......................................................................ugagaNcauugugaaaacu...................   1        1    Fm3
......................................................................ugagaucauuguAaaaacu...................   2        1    Fm3
......................................................................uCagaucauugugaaaacu...................   2        1    Fm3
......................................................................ugagaucauuguCaaaacu...................   1        1    Fm3
......................................................................ugagaucauugugaaaGcu...................   31       1    Fm3
......................................................................ugagaucauugugaaaaGu...................   108      1    Fm3
......................................................................ugagauUauugugaaaacu...................   8        1    Fm3
......................................................................ugagaucGuugugaaaacu...................   9        1    Fm3
......................................................................ugagaucauugugaaaacG...................   32       1    Fm3
......................................................................uAagaucauugugaaaacu...................   8        1    Fm3
......................................................................ugagaucauugAgaaaacu...................   1        1    Fm3
......................................................................ugagauAauugugaaaacu...................   2        1    Fm3
......................................................................ugagaucauuguUaaaacu...................   1        1    Fm3
......................................................................ugagaucauugugaaaacu...................   20479    0    Fm3
......................................................................ugNgaucauugugaaaacu...................   2        1    Fm3
......................................................................ugagaucauuUugaaaacu...................   27       1    Fm3
......................................................................ugagaucauugugaaaaAu...................   288      1    Fm3
......................................................................ugagUucauugugaaaacu...................   5        1    Fm3
......................................................................ugaAaucauugugaaaacu...................   4        1    Fm3
......................................................................ugaAaucauugugaaaacug..................   36       1    Fm3
......................................................................ugagaucauugugaaUacug..................   5        1    Fm3
......................................................................ugagaNcauugugaaaacug..................   27       1    Fm3
......................................................................ugagaucauugugaaaacuC..................   9        1    Fm3
......................................................................ugagaucauAgugaaaacug..................   8        1    Fm3
......................................................................ugagaucauugugaUaacug..................   19       1    Fm3
......................................................................ugagaucauuCugaaaacug..................   146      1    Fm3
......................................................................ugagaucauugugaaGacug..................   19       1    Fm3
......................................................................uCagaucauugugaaaacug..................   20       1    Fm3
......................................................................Cgagaucauugugaaaacug..................   20       1    Fm3
......................................................................ugagaucauugugaaaaUug..................   673      1    Fm3
......................................................................ugagaucauugCgaaaacug..................   26       1    Fm3
......................................................................ugagaucauugugaaaCcug..................   221      1    Fm3
......................................................................ugagUucauugugaaaacug..................   14       1    Fm3
......................................................................ugagaucauugAgaaaacug..................   12       1    Fm3
......................................................................ugagaucauuguUaaaacug..................   20       1    Fm3
......................................................................ugagaGcauugugaaaacug..................   15       1    Fm3
......................................................................uAagaucauugugaaaacug..................   43       1    Fm3
......................................................................ugagCucauugugaaaacug..................   25       1    Fm3
......................................................................ugaCaucauugugaaaacug..................   10       1    Fm3
......................................................................ugagaucaCugugaaaacug..................   13       1    Fm3
......................................................................ugagaucauuguAaaaacug..................   18       1    Fm3
......................................................................ugCgaucauugugaaaacug..................   15       1    Fm3
......................................................................ugagaCcauugugaaaacug..................   23       1    Fm3
......................................................................ugagaucaAugugaaaacug..................   6        1    Fm3
......................................................................ugagaucauuUugaaaacug..................   92       1    Fm3
......................................................................ugagaucauugugGaaacug..................   20       1    Fm3
......................................................................ugGgaucauugugaaaacug..................   56       1    Fm3
......................................................................ugagaucauugugaaaacuA..................   550      1    Fm3
......................................................................ugagaucauugugaaaaAug..................   1192     1    Fm3
......................................................................ugagaucauCgugaaaacug..................   11       1    Fm3
......................................................................ugagaucauugugaaaacug..................   142469   0    Fm3
......................................................................ugagaucauugugaaaGcug..................   181      1    Fm3
......................................................................ugagaucauugugaaCacug..................   3        1    Fm3
......................................................................ugagaucauugugaaaaGug..................   630      1    Fm3
......................................................................Ngagaucauugugaaaacug..................   66       1    Fm3
......................................................................ugagauGauugugaaaacug..................   7        1    Fm3
......................................................................ugagauNauugugaaaacug..................   4        1    Fm3
......................................................................ugagaucauugugaaaacAg..................   4        1    Fm3
```

Star **Mature**

```
uguuggcuguaaugaugaccuucagacgaaacugguuuucauaaugaccuuccagauuguugugcuauucugagaucauugugaaaacugauuccguugcccccaauc
......................................................................ugagaucauugugaGaacug..................  23      1   Fm3
......................................................................ugagaucauugugaCaacug..................  1       1   Fm3
......................................................................ugagaucaGugugaaaacug..................  9       1   Fm3
......................................................................Ggagaucauugugaaaacug..................  4       1   Fm3
......................................................................ugagaucauGgugaaaacug..................  8       1   Fm3
......................................................................ugagaucUuugugaaaacug..................  23      1   Fm3
......................................................................uUagaucauugugaaaacug..................  42      1   Fm3
......................................................................ugNgaucauugugaaaacug..................  1       1   Fm3
......................................................................ugaUaucauugugaaaacug..................  13      1   Fm3
......................................................................ugagaucauugugaaaUcug..................  270     1   Fm3
......................................................................ugagaucauugugUaaacug..................  33      1   Fm3
......................................................................ugUgaucauugugaaaacug..................  24      1   Fm3
......................................................................ugagaucauugGgaaaacug..................  5       1   Fm3
......................................................................ugagGucauugugaaaacug..................  43      1   Fm3
......................................................................ugagauUauugugaaaacug..................  58      1   Fm3
......................................................................uNagaucauugugaaaacug..................  4       1   Fm3
......................................................................ugagauAauugugaaaacug..................  8       1   Fm3
......................................................................ugagaucauugugaaaacGg..................  4       1   Fm3
......................................................................ugagaucGuugugaaaacug..................  22      1   Fm3
......................................................................ugagaucauuguCaaaacug..................  6       1   Fm3
......................................................................ugagaAcauugugaaaacug..................  9       1   Fm3
......................................................................ugagaucauugugaaaaCug..................  27      1   Fm3
......................................................................ugagaucauugugaaaacuU..................  124     1   Fm3
......................................................................Agagaucauugugaaaacug..................  13      1   Fm3
......................................................................ugagaucauugugCaaacug..................  7       1   Fm3
......................................................................ugagaucauuAugaaaacug..................  276     1   Fm3
......................................................................uUagaucauugugaaaacuga.................  34      1   Fm3
......................................................................uCagaucauugugaaaacuga.................  16      1   Fm3
......................................................................ugagaucauugugaaaCcuga.................  166     1   Fm3
......................................................................ugagauUauugugaaaacuga.................  32      1   Fm3
......................................................................ugagaucauuguAaaaacuga.................  8       1   Fm3
......................................................................ugaUaucauugugaaaacuga.................  14      1   Fm3
......................................................................ugagaucaCugugaaaacuga.................  7       1   Fm3
......................................................................ugagaucUuugugaaaacuga.................  12      1   Fm3
......................................................................ugagaucauugugaaaacuga.................  99043   0   Fm3
......................................................................ugagUucauugugaaaacuga.................  15      1   Fm3
......................................................................ugagaucauCgugaaaacuga.................  10      1   Fm3
......................................................................uAagaucauugugaaaacuga.................  58      1   Fm3
......................................................................ugagaCcauugugaaaacuga.................  8       1   Fm3
......................................................................ugagaucauugugGaaacuga.................  16      1   Fm3
......................................................................ugagaucauuCugaaaacuga.................  101     1   Fm3
......................................................................ugagaucaGugugaaaacuga.................  7       1   Fm3
......................................................................ugagaucauugugaaaacuUa.................  15      1   Fm3
......................................................................ugaCaucauugugaaaacuga.................  5       1   Fm3
......................................................................ugagauNauugugaaaacuga.................  10      1   Fm3
......................................................................ugagaucCuugugaaaacuga.................  1       1   Fm3
......................................................................ugagaucauugugaaUacuga.................  8       1   Fm3
......................................................................ugagaucauugugaaaaAuga.................  798     1   Fm3
......................................................................ugCgaucauugugaaaacuga.................  14      1   Fm3
......................................................................ugagaAcauugugaaaacuga.................  5       1   Fm3
......................................................................ugagauGauugugaaaacuga.................  7       1   Fm3
......................................................................ugagaucauugugaaaacugU.................  1359    1   Fm3
......................................................................ugagaucauugGgaaaacuga.................  2       1   Fm3
......................................................................ugGgaucauugugaaaacuga.................  45      1   Fm3
......................................................................ugagaNcauugugaaaacuga.................  14      1   Fm3
......................................................................ugagaucauugugCaaacuga.................  5       1   Fm3
......................................................................ugagaucauugugaaaacGga.................  5       1   Fm3
......................................................................ugagaucauugugaaaacugC.................  3       1   Fm3
......................................................................ugagaucauugAgaaaacuga.................  13      1   Fm3
......................................................................Ggagaucauugugaaaacuga.................  2       1   Fm3
......................................................................ugagaucauuguUaaaacuga.................  15      1   Fm3
......................................................................ugagaucauugCgaaaacuga.................  13      1   Fm3
......................................................................ugagaucauuAugaaaacuga.................  192     1   Fm3
......................................................................ugagaucauugugaaGacuga.................  13      1   Fm3
......................................................................ugagGucauugugaaaacuga.................  21      1   Fm3
......................................................................ugagauAauugugaaaacuga.................  2       1   Fm3
......................................................................ugUgaucauugugaaaacuga.................  14      1   Fm3
......................................................................Agagaucauugugaaaacuga.................  5       1   Fm3
......................................................................ugagaucauuUugaaaacuga.................  71      1   Fm3
......................................................................ugagaucauugugaaaacAga.................  4       1   Fm3
```

Star Mature

```
uguuggcuguaaugaugaccuucagacgaaacugguuuucauaaaugaccuuccagauuguugugcuauucugagaucauugugaaaacugauuccguugcccccaauc
.......................................................................ugagaGcauugugaaaacuga.................  5      1    Fm3
.......................................................................Cgagaucauugugaaaacuga.................  14     1    Fm3
.......................................................................ugagaucauugugaaaacugG.................  10     1    Fm3
.......................................................................ugagaucauugugaaaaUuga.................  500    1    Fm3
.......................................................................ugaAaucauugugaaaacuga.................  16     1    Fm3
.......................................................................Ngagaucauugugaaaacuga.................  40     1    Fm3
.......................................................................ugagaucauugugaaaaGuga.................  459    1    Fm3
.......................................................................ugagaucauugugaaCacuga.................  1      1    Fm3
.......................................................................ugagaucauugugaaaGcuga.................  116    1    Fm3
.......................................................................ugagaucauAgugaaaacuga.................  6      1    Fm3
.......................................................................ugagaucauugugaaaUcuga.................  177    1    Fm3
.......................................................................ugagaucauugugaaaacuAa.................  11     1    Fm3
.......................................................................ugagaucauugugaaaaCga.................  13     1    Fm3
.......................................................................ugagCucauugugaaaacuga.................  21     1    Fm3
.......................................................................ugagaucauugugaUaacuga.................  6      1    Fm3
.......................................................................ugagaucaAugugaaaacuga.................  1      1    Fm3
.......................................................................ugagaucauugugaGaacuga.................  17     1    Fm3
.......................................................................ugagaucGuugugaaaacuga.................  26     1    Fm3
.......................................................................ugagaucauugugaCaacuga.................  4      1    Fm3
.......................................................................ugagaucauuguCaaaacuga.................  16     1    Fm3
.......................................................................ugagaucauugugUaaacuga.................  16     1    Fm3
.......................................................................ugagaucauGgugaaaacuga.................  9      1    Fm3
.......................................................................ugagaucauugugaaaacuCa.................  5      1    Fm3
.......................................................................ugagaucauugugaGaacugau................  13     1    Fm3
.......................................................................ugagaucauugugaaaacuCau................  3      1    Fm3
.......................................................................ugagaucauugugaaaacuAau................  6      1    Fm3
.......................................................................ugagaucauugAgaaaacugau................  7      1    Fm3
.......................................................................ugagaucauugugaaUacugau................  4      1    Fm3
.......................................................................ugagaucauugugaaaacuUau................  11     1    Fm3
.......................................................................ugagaucauugugaaaacugUu................  15     1    Fm3
.......................................................................ugagaucauugugaaaacugaG................  1      1    Fm3
.......................................................................ugagUucauugugaaaacugau................  11     1    Fm3
.......................................................................ugagaucaGugugaaaacugau................  3      1    Fm3
.......................................................................ugagaucauugugaaaacugGu................  5      1    Fm3
.......................................................................uUagaucauugugaaaacugau................  21     1    Fm3
.......................................................................ugagaucauugugaaaUcugau................  125    1    Fm3
.......................................................................ugagaucauugugaaaacugaA................  714    1    Fm3
.......................................................................ugagauAauugugaaaacugau................  1      1    Fm3
.......................................................................ugagaucauuAugaaaacugau................  117    1    Fm3
.......................................................................ugagaucauugugaaaGcugau................  75     1    Fm3
.......................................................................ugagaucauugugaaaaAugau................  510    1    Fm3
.......................................................................ugaUaucauugugaaaacugau................  7      1    Fm3
.......................................................................ugagaucauugugaaCacugau................  2      1    Fm3
.......................................................................ugagaucauugugUaaacugau................  10     1    Fm3
.......................................................................ugagaucauugugaaaaGugau................  244    1    Fm3
.......................................................................ugagaucauugugCaaacugau................  5      1    Fm3
.......................................................................Ngagaucauugugaaaacugau................  31     1    Fm3
.......................................................................ugagaucauugugGaaacugau................  10     1    Fm3
.......................................................................ugGgaucauugugaaaacugau................  22     1    Fm3
.......................................................................ugaAaucauugugaaaacugau................  10     1    Fm3
.......................................................................ugagaucauugugaaGacugau................  5      1    Fm3
.......................................................................ugagaucauuguAaaaacugau................  7      1    Fm3
.......................................................................ugagaucauugugaaaacugaC................  6      1    Fm3
.......................................................................uNagaucauugugaaaacugau................  3      1    Fm3
.......................................................................ugagaucauugugaaaacugau................  58032  0    Fm3
.......................................................................Agagaucauugugaaaacugau................  3      1    Fm3
.......................................................................ugagaucauuCugaaaacugau................  69     1    Fm3
.......................................................................ugagaucUuugugaaaacugau................  9      1    Fm3
.......................................................................ugagaucauuguCaaaacugau................  7      1    Fm3
.......................................................................ugagCucauugugaaaacugau................  7      1    Fm3
.......................................................................ugCgaucauugugaaaacugau................  4      1    Fm3
.......................................................................ugUgaucauugugaaaacugau................  12     1    Fm3
.......................................................................ugagaucauugugaaaaCgau................  5      1    Fm3
.......................................................................Cgagaucauugugaaaacugau................  6      1    Fm3
.......................................................................ugagauNauugugaaaacugau................  4      1    Fm3
.......................................................................ugaCaucauugugaaaacugau................  6      1    Fm3
.......................................................................ugagaucauugugaCaacugau................  1      1    Fm3
.......................................................................ugagaucauugugaaaaUugau................  280    1    Fm3
.......................................................................ugagaucauGgugaaaacugau................  4      1    Fm3
.......................................................................ugagaucauuUugaaaacugau................  43     1    Fm3
```

Star Mature

uguuggcuguaaugaugaccuucagacgaaacugguuuucauaaugaccuuccagauuguugugcuauucugagaucauugugaaaacugauuccguugcccccaauc			
....ugagaucGuugugaaaacugau................	18	1	Fm3
....uCagaucauugugaaaacugau................	3	1	Fm3
....uAagaucauugugaaaacugau................	25	1	Fm3
....ugagaucauugugaaaCcugau................	85	1	Fm3
....ugagauUauugugaaaacugau................	29	1	Fm3
....ugagaucauugugaaaacugCu................	1	1	Fm3
....ugagaCcauugugaaaacugau................	3	1	Fm3
....Ggagaucauugugaaaacugau................	1	1	Fm3
....ugagGucauugugaaaacugau................	8	1	Fm3
....ugagaucCuugugaaaacugau................	1	1	Fm3
....ugagaucauugugaaaacGgau................	2	1	Fm3
....ugagaucaCugugaaaacugau................	7	1	Fm3
....ugagaucauuguUaaaacugau................	9	1	Fm3
....ugagaucaAugugaaaacugau................	5	1	Fm3
....ugagaAcauugugaaaacugau................	4	1	Fm3
....ugagaucauugCgaaaacugau................	14	1	Fm3
....ugagaucauugGgaaaacugau................	7	1	Fm3
....ugagaucauAgugaaaacugau................	3	1	Fm3
....ugagaNcauugugaaaacugau................	10	1	Fm3
....ugagauGauugugaaaacugau................	3	1	Fm3
....ugagaucauugugaaaacAgau................	6	1	Fm3
....ugagaucauCgugaaaacugau................	5	1	Fm3
....ugagaucauugugaaaUcugauu...............	108	1	Fm3
....uUagaucauugugaaaacugauu...............	16	1	Fm3
....ugagaAcauugugaaaacugauu...............	8	1	Fm3
....ugagaucauugugGaaacugauu...............	10	1	Fm3
....ugagaucauugugaaaacugUuu...............	4	1	Fm3
....ugagaucauugugaaaCcugauu...............	86	1	Fm3
....ugagUucauugugaaaacugauu...............	8	1	Fm3
....ugGgaucauugugaaaacugauu...............	19	1	Fm3
....ugagaucauCgugaaaacugauu...............	8	1	Fm3
....Cgagaucauugugaaaacugauu...............	15	1	Fm3
....ugagaucauugugaaaaUugauu...............	284	1	Fm3
....uAagaucauugugaaaacugauu...............	25	1	Fm3
....ugagaucauugugaaaaAugauu...............	501	1	Fm3
....ugagauGauugugaaaacugauu...............	5	1	Fm3
....ugUgaucauugugaaaacugauu...............	12	1	Fm3
....ugagaucauugugaaaacugaCu...............	2	1	Fm3
....ugagaucauugugaUaacugauu...............	6	1	Fm3
....ugagaucauugugaaGacugauu...............	13	1	Fm3
....ugagaucauugugaaaacugauA...............	221	1	Fm3
....ugagaucauugugaaaacugauG...............	3	1	Fm3
....ugagaucauugugaaaacugaGu...............	2	1	Fm3
....ugCgaucauugugaaaacugauu...............	7	1	Fm3
....ugagauNauugugaaaacugauu...............	7	1	Fm3
....ugagaucauugCgaaaacugauu...............	11	1	Fm3
....ugagaucauGgugaaaacugauu...............	4	1	Fm3
....ugagaucauugugaaaacuUauu...............	9	1	Fm3
....ugaCaucauugugaaaacugauu...............	5	1	Fm3
....ugagaucauugugUaaacugauu...............	12	1	Fm3
....Ngagaucauugugaaaacugauu...............	26	1	Fm3
....ugagaGcauugugaaaacugauu...............	3	1	Fm3
....ugagaucauugugaaCacugauu...............	1	1	Fm3
....ugagaucauugugaaaGcugauu...............	74	1	Fm3
....ugagaNcauugugaaaacugauu...............	7	1	Fm3
....ugagaucauuguAaaaacugauu...............	11	1	Fm3
....ugaUaucauugugaaaacugauu...............	5	1	Fm3
....ugagaucauugugaGaacugauu...............	16	1	Fm3
....ugagaucUuugugaaaacugauu...............	8	1	Fm3
....ugagaucauAgugaaaacugauu...............	4	1	Fm3
....ugagaucauugugaaaacugauu...............	58028	0	Fm3
....ugagaucauuUugaaaacugauu...............	55	1	Fm3
....ugagaucaGugugaaaacugauu...............	4	1	Fm3
....Ggagaucauugugaaaacugauu...............	2	1	Fm3
....ugagaucauugugaaaacuAauu...............	4	1	Fm3
....ugagaucauugAgaaaacugauu...............	7	1	Fm3
....ugagaCcauugugaaaacugauu...............	5	1	Fm3
....ugagaucauugugaaaaCCgauu...............	10	1	Fm3
....ugagauAauugugaaaacugauu...............	3	1	Fm3
....ugagaucGuugugaaaacugauu...............	14	1	Fm3

```
                                    Star                               Mature
uguuggcuguaaugaugaccuucagacgaaacugguuuucauaaugaccuuccagauuguugugcuauucugagaucauugugaaaacugauuccguugcccccaauc
......................................................................uCagaucauugugaaaacugauu...............    7      1      Fm3
......................................................................ugaAaucauugugaaaacugauu...............    7      1      Fm3
......................................................................ugagaucauuCugaaaacugauu...............    55     1      Fm3
......................................................................ugagaucauugugaaaaGugauu...............    262    1      Fm3
......................................................................ugagauUauugugaaaacugauu...............    17     1      Fm3
......................................................................ugagaucauuguUaaaacugauu...............    8      1      Fm3
......................................................................ugagaucauugugaaaacAgauu...............    2      1      Fm3
......................................................................ugagaucauugugaaaacugauC...............    3      1      Fm3
......................................................................ugagaucauuguCaaaacugauu...............    4      1      Fm3
......................................................................ugNgaucauugugaaaacugauu...............    1      1      Fm3
......................................................................ugagaucauuAugaaaacugauu...............    123    1      Fm3
......................................................................ugagaucauugGgaaaacugauu...............    1      1      Fm3
......................................................................ugagaucaAugugaaaacugauu...............    2      1      Fm3
......................................................................ugagGucauugugaaaacugauu...............    14     1      Fm3
......................................................................Agagaucauugugaaaacugauu...............    6      1      Fm3
......................................................................ugagaucauugugaaaacugGuu...............    7      1      Fm3
......................................................................ugagaucauugugaaUacugauu...............    7      1      Fm3
......................................................................ugagaucauugugCaaacugauu...............    4      1      Fm3
......................................................................ugagaucauugugaaaacugCuu...............    1      1      Fm3
......................................................................ugagaucauugugaaaacuCauu...............    4      1      Fm3
......................................................................ugagCucauugugaaaacugauu...............    13     1      Fm3
......................................................................ugagaucauugugaaaacugaAu...............    27     1      Fm3
......................................................................ugagaucaCugugaaaacugauu...............    2      1      Fm3
......................................................................ugagaucauugugaaaacugauuU..............    4874   1      Fm3
......................................................................ugagaucauugugaaaacugauuG..............    2      1      Fm3
......................................................................ugagaucauugugaaaacugauuA..............    138    1      Fm3
......................................................................ugagaucauugugaaaacugauuc..............    6      0      Fm3
.......................................................................Uagaucauugugaaaacu...................    1      1      Fm3
.......................................................................gagaucauuAugaaaacug..................    1      1      Fm3
.......................................................................gagaucauugugaaaacug..................    95     0      Fm3
.......................................................................gagaucauuUugaaaacug..................    2      1      Fm3
.......................................................................Uagaucauugugaaaacug..................    2      1      Fm3
.......................................................................gagaucauugugaaaacuga.................    8      0      Fm3
.......................................................................gagaucauugugaaaacugU.................    1      1      Fm3
.......................................................................gagaucauugugaaaacugaA................    1      1      Fm3
.......................................................................gagaucauugugaaaacugau................    6      0      Fm3
.......................................................................Uagaucauugugaaaacugau................    1      1      Fm3
.......................................................................gagaucauugugaaaacugauu...............    14     0      Fm3
.......................................................................gagaucauugugaaaacugUuu...............    1      1      Fm3
.......................................................................Uagaucauugugaaaacugauu...............    2      1      Fm3
.......................................................................gagaucauugugaaaacugauuU..............    1      1      Fm3
........................................................................Ugaucauugugaaaacug..................    6      1      Fm3
........................................................................Ugaucauugugaaaacugau................    1      1      Fm3
........................................................................Ugaucauugugaaaacugauu...............    4      1      Fm3

...............................cugguuuucauaaugaccuucc.......................................................    4      0      Mm1
...............................cugguuuucauaaugaUcuuccag.....................................................    1      1      Mm1
...............................cugguuuucauaaugaccuuccag.....................................................    12     0      Mm1
...................................uuuucauaaugaccuuccaga....................................................    1      0      Mm1
....................................uuucauaaugaccuuccaga....................................................    2      0      Mm1
..................................................................auucugagaucauugugaaaa.....................    7      0      Mm1
..................................................................auucugagaucauugugUaaa.....................    1      1      Mm1
...................................................................uucugagaucauugugCaaacu...................    1      1      Mm1
...................................................................uucugagaucauugugUaaacu...................    1      1      Mm1
...................................................................uucugagaucauugugaaaacu...................    126    0      Mm1
...................................................................uucugagaucauugugaCaacu...................    2      1      Mm1
...................................................................uucugagaucauuguUaaaacu...................    1      1      Mm1
...................................................................uucugagaucauugugaUaacu...................    1      1      Mm1
...................................................................uucugagaucauugugaaaacuA..................    5      1      Mm1
.....................................................................cugagaucauugugaaaacug..................    6      0      Mm1
......................................................................ugagaucauugugaaaaG....................    45     1      Mm1
......................................................................ugagaucauugugaaaaU....................    2      1      Mm1
......................................................................ugagaucauugugaaaGc....................    2      1      Mm1
......................................................................ugagaucauugugaaaaA....................    42     1      Mm1
......................................................................ugagaucauugugaaaac....................    71     0      Mm1
......................................................................ugagaucGuugugaaaacu...................    4      1      Mm1
......................................................................Cgagaucauugugaaaacu...................    1      1      Mm1
......................................................................ugagaucauuCugaaaacu...................    1      1      Mm1
......................................................................ugagaucauuUugaaaacu...................    2      1      Mm1
......................................................................ugagaucUuugugaaaacu...................    1      1      Mm1
```

Star / Mature			
uguuggcuguaaugaugaccuucagacgaaacugguuuucauaaugaccuuccagauuguugugcuauucugagaucauugugaaaacugauuccguugcccccaauc			
....ugUgaucauugugaaaacu...................	3	1	Mm1
....uAagaucauugugaaaacu...................	1	1	Mm1
....ugagaucauugugaaaUcu...................	5	1	Mm1
....ugagaucauugugaaaaAu...................	28	1	Mm1
....ugagaucauugugaaaaGu...................	17	1	Mm1
....ugagaucauugugaaaGcu...................	2	1	Mm1
....ugagaAcauugugaaaacu...................	1	1	Mm1
....ugagaucauugugaaaCcu...................	5	1	Mm1
....ugagaucauugugaaaaUu...................	14	1	Mm1
....ugGgaucauugugaaaacu...................	1	1	Mm1
....ugCgaucauugugaaaacu...................	1	1	Mm1
....ugagaucauGgugaaaacu...................	1	1	Mm1
....Ngagaucauugugaaaacu...................	3	1	Mm1
....ugagaucauugugaaaacu...................	2414	0	Mm1
....ugagCucauugugaaaacu...................	2	1	Mm1
....ugagaucauugugaaaaGug..................	122	1	Mm1
....ugaAaucauugugaaaacug..................	2	1	Mm1
....ugagaucauugugUaaacug..................	4	1	Mm1
....ugagaucaCugugaaaacug..................	3	1	Mm1
....ugagaucauGgugaaaacug..................	5	1	Mm1
....ugagGucauugugaaaacug..................	2	1	Mm1
....ugagaucauugugaGaacug..................	3	1	Mm1
....ugGgaucauugugaaaacug..................	18	1	Mm1
....ugUgaucauugugaaaacug..................	6	1	Mm1
....ugagaucauuCugaaaacug..................	22	1	Mm1
....ugagaucauugugaaaGcug..................	35	1	Mm1
....ugagaucaAugugaaaacug..................	1	1	Mm1
....ugagaucauugugaaaacuC..................	1	1	Mm1
....ugagUucauugugaaaacug..................	3	1	Mm1
....ugagaucauugugaaGacug..................	4	1	Mm1
....Cgagaucauugugaaaacug..................	6	1	Mm1
....ugagaucGuugugaaaacug..................	17	1	Mm1
....ugagaucauugugGaaacug..................	6	1	Mm1
....ugagaucauugugaaaUcug..................	57	1	Mm1
....Ggagaucauugugaaaacug..................	1	1	Mm1
....ugagaucauuUugaaaacug..................	30	1	Mm1
....ugagaucauuguUaaaacug..................	4	1	Mm1
....ugagaucauugugaaCacug..................	1	1	Mm1
....ugagauUauugugaaaacug..................	6	1	Mm1
....uAagaucauugugaaaacug..................	11	1	Mm1
....uCagaucauugugaaaacug..................	2	1	Mm1
....ugagaucauugCgaaaacug..................	4	1	Mm1
....ugagaucauugugaUaacug..................	2	1	Mm1
....ugagauGauugugaaaacug..................	1	1	Mm1
....ugagaucauugugaaaaAug..................	272	1	Mm1
....ugagaNcauugugaaaacug..................	7	1	Mm1
....ugagaucauuAugaaaacug..................	80	1	Mm1
....ugaUaucauugugaaaacug..................	1	1	Mm1
....ugagaucaGugugaaaacug..................	3	1	Mm1
....ugagaucauuguAaaaacug..................	9	1	Mm1
....ugagaucauugAgaaaacug..................	2	1	Mm1
....ugagaucauugugaaaacCg..................	1	1	Mm1
....ugagCucauugugaaaacug..................	13	1	Mm1
....ugagaucauugugaaaacuU..................	27	1	Mm1
....ugagaucauuguCaaaacug..................	1	1	Mm1
....Ngagaucauugugaaaacug..................	18	1	Mm1
....ugagaAcauugugaaaacug..................	1	1	Mm1
....ugagaucauAgugaaaacug..................	2	1	Mm1
....ugagauAauugugaaaacug..................	4	1	Mm1
....ugagaucauugGgaaaacug..................	1	1	Mm1
....Agagaucauugugaaaacug..................	2	1	Mm1
....ugagaucauugugaaUacug..................	3	1	Mm1
....ugagaGcauugugaaaacug..................	2	1	Mm1
....uUagaucauugugaaaacug..................	7	1	Mm1
....ugagaucauugugaaaacuA..................	44	1	Mm1
....ugagaucauugugaaaacug..................	29250	0	Mm1
....ugagaucauugugaaaacGg..................	14	1	Mm1
....ugagaucauugugaaaCcug..................	58	1	Mm1
....ugagaCcauugugaaaacug..................	1	1	Mm1
....ugagaucauCgugaaaacug..................	6	1	Mm1

Star **Mature**

```
uguuggcuguaaugaugaccuucagacgaaacugguuuucauaaugaccuuccagauuguugugcuauucugagaucauugugaaaacugauuccguugcccccaauc
......................................................................ugagaucauugugaaaaUug..................    152    1  Mm1
......................................................................ugagaucUuugugaaaacug..................    3      1  Mm1
......................................................................ugagaucauugugaGaacuga.................    1      1  Mm1
......................................................................ugagGucauugugaaaacuga.................    3      1  Mm1
......................................................................ugagaucauugugaaaaAuga.................    103    1  Mm1
......................................................................ugagaucauugugaaaaGuga.................    51     1  Mm1
......................................................................ugagaucauugAgaaaacuga.................    1      1  Mm1
......................................................................Ngagaucauugugaaaacuga.................    9      1  Mm1
......................................................................uCagaucauugugaaaacuga.................    2      1  Mm1
......................................................................ugagaucauAgugaaaacuga.................    1      1  Mm1
......................................................................ugagaucauugugaaaUcuga.................    27     1  Mm1
......................................................................ugagaucauugugaaaacuCa.................    2      1  Mm1
......................................................................ugCgaucauugugaaaacuga.................    1      1  Mm1
......................................................................uAagaucauugugaaaacuga.................    9      1  Mm1
......................................................................ugagaucauugugaaaacuga.................    12838  0  Mm1
......................................................................uNagaucauugugaaaacuga.................    1      1  Mm1
......................................................................ugagaucCuugugaaaacuga.................    2      1  Mm1
......................................................................ugaUaucauugugaaaacuga.................    1      1  Mm1
......................................................................ugagaucauugugaaaacugU.................    267    1  Mm1
......................................................................ugagaucauugugaaaCcuga.................    17     1  Mm1
......................................................................ugagaucauugugUaaacuga.................    3      1  Mm1
......................................................................ugagaucauugugaaaacugG.................    39     1  Mm1
......................................................................ugagaucauugugaCaacuga.................    1      1  Mm1
......................................................................ugagaAcauugugaaaacuga.................    3      1  Mm1
......................................................................ugUgaucauugugaaaacuga.................    3      1  Mm1
......................................................................ugagaucauugugaaaacugC.................    1      1  Mm1
......................................................................ugagaucGuugugaaaacuga.................    1      1  Mm1
......................................................................ugagaucauugugaaaaUuga.................    61     1  Mm1
......................................................................ugagauGauugugaaaacuga.................    1      1  Mm1
......................................................................ugagaucauugugaaaacuAa.................    1      1  Mm1
......................................................................ugagaGcauugugaaaacuga.................    1      1  Mm1
......................................................................ugagUucauugugaaaacuga.................    2      1  Mm1
......................................................................ugagaucauuUugaaaacuga.................    5      1  Mm1
......................................................................ugagaNcauugugaaaacuga.................    1      1  Mm1
......................................................................ugagauUauugugaaaacuga.................    2      1  Mm1
......................................................................ugagaucauugugaaaacCga.................    1      1  Mm1
......................................................................ugaAaucauugugaaaacuga.................    1      1  Mm1
......................................................................ugagaucauugugCaaacuga.................    1      1  Mm1
......................................................................ugagaucauCgugaaaacuga.................    2      1  Mm1
......................................................................ugagaucauuCugaaaacuga.................    17     1  Mm1
......................................................................Cgagaucauugugaaaacuga.................    5      1  Mm1
......................................................................Agagaucauugugaaaacuga.................    1      1  Mm1
......................................................................ugagaucauugugaaGacuga.................    1      1  Mm1
......................................................................ugagaucaCugugaaaacuga.................    4      1  Mm1
......................................................................ugagaucauugugaaaGcuga.................    13     1  Mm1
......................................................................ugagCucauugugaaaacuga.................    5      1  Mm1
......................................................................ugagaucauugugGaaacuga.................    1      1  Mm1
......................................................................ugagaucauuguAaaacuga.................    2      1  Mm1
......................................................................ugGgaucauugugaaaacuga.................    5      1  Mm1
......................................................................ugagaucauuAugaaaacuga.................    22     1  Mm1
......................................................................ugagaucauugCgaaaacuga.................    7      1  Mm1
......................................................................ugagaucauugugaaaGcugau................    12     1  Mm1
......................................................................ugagaucauugugaaaacuUau................    4      1  Mm1
......................................................................ugagaucauCgugaaaacugau................    9      1  Mm1
......................................................................ugagaucauugugaaaacugUu................    6      1  Mm1
......................................................................ugagCucauugugaaaacugau................    3      1  Mm1
......................................................................ugagaucauugugGaaacugau................    1      1  Mm1
......................................................................ugagaucauugugaUaacugau................    1      1  Mm1
......................................................................uCagaucauugugaaaacugau................    2      1  Mm1
......................................................................ugagaucauugugaaaaAugau................    80     1  Mm1
......................................................................ugagaucauuUugaaaacugau................    8      1  Mm1
......................................................................ugGgaucauugugaaaacugau................    6      1  Mm1
......................................................................ugUgaucauugugaaaacugau................    1      1  Mm1
......................................................................uAagaucauugugaaaacugau................    1      1  Mm1
......................................................................ugagaucauugugaaaaGugau................    32     1  Mm1
......................................................................ugagaucCuugugaaaacugau................    1      1  Mm1
......................................................................ugagaucauugugaaaUcugau................    10     1  Mm1
......................................................................ugagaucaGugugaaaacugau................    1      1  Mm1
......................................................................ugagaucauugugaaaacCgau................    1      1  Mm1
......................................................................ugagauUauugugaaaacugau................    2      1  Mm1
```

Star **Mature**

```
uguuggcuguaaugaugaccuucagacgaaacugguuuucauaaugaccuuccagauuguugugcuauucugagaucauugugaaaacugauuccguugcccccaauc
......................................................................ugagaucauuCugaaaacugau................ 7 1 Mm1
......................................................................ugagaucauugugaaaCcugau................ 17 1 Mm1
......................................................................ugagaucGuugugaaaacugau................ 6 1 Mm1
......................................................................ugagaucauugCgaaaacugau................ 2 1 Mm1
......................................................................ugagaucauuAugaaaacugau................ 21 1 Mm1
......................................................................ugagaucauugugCaaacugau................ 1 1 Mm1
......................................................................Ngagaucauugugaaaacugau................ 3 1 Mm1
......................................................................ugagaucauugugaGaacugau................ 2 1 Mm1
......................................................................ugagaCcauugugaaaacugau................ 1 1 Mm1
......................................................................ugagaucauugugaaaacugaA................ 117 1 Mm1
......................................................................ugagaucauugugUaaacugau................ 2 1 Mm1
......................................................................ugagaucaCugugaaaacugau................ 1 1 Mm1
......................................................................ugagaucauugugaaaacugCu................ 1 1 Mm1
......................................................................ugCgaucauugugaaaacugau................ 1 1 Mm1
......................................................................ugagaucauugugaaaaUugau................ 53 1 Mm1
......................................................................ugagaucUuugugaaaacugau................ 1 1 Mm1
......................................................................ugagaucauAgugaaaacugau................ 1 1 Mm1
......................................................................ugagaNcauugugaaaacugau................ 3 1 Mm1
......................................................................ugagaucauugugaaGacugau................ 1 1 Mm1
......................................................................ugagaucauugugaaaacugau................ 9187 0 Mm1
......................................................................ugagCucauugugaaaacugauu............... 7 1 Mm1
......................................................................ugagaucauugugaaaCcugauu............... 18 1 Mm1
......................................................................ugagaucauugugGaaacugauu............... 1 1 Mm1
......................................................................ugagaucauuUugaaaacugauu............... 6 1 Mm1
......................................................................ugagaucauugugaaGacugauu............... 3 1 Mm1
......................................................................ugagaNcauugugaaaacugauu............... 2 1 Mm1
......................................................................ugagaucauuCugaaaacugauu............... 7 1 Mm1
......................................................................ugagaucauugugaaaacugaAu............... 10 1 Mm1
......................................................................ugUgaucauugugaaaacugauu............... 1 1 Mm1
......................................................................ugagaucauugugaaaacugauA............... 57 1 Mm1
......................................................................ugaAaucauugugaaaacugauu............... 2 1 Mm1
......................................................................ugagaucauugugaaaacugGuu............... 2 1 Mm1
......................................................................ugagaucauugCgaaaacugauu............... 1 1 Mm1
......................................................................ugagaucauGgugaaaacugauu............... 1 1 Mm1
......................................................................ugagaucauugugaaaacugauC............... 4 1 Mm1
......................................................................ugagaucauugugaaaacugUuu............... 2 1 Mm1
......................................................................ugagaucauugugaaaaGugauu............... 43 1 Mm1
......................................................................ugagGucauugugaaaacugauu............... 1 1 Mm1
......................................................................ugagaucauugugaGaacugauu............... 1 1 Mm1
......................................................................ugagaAcauugugaaaacugauu............... 2 1 Mm1
......................................................................uCagaucauugugaaaacugauu............... 1 1 Mm1
......................................................................ugGgaucauugugaaaacugauu............... 10 1 Mm1
......................................................................uAagaucauugugaaaacugauu............... 2 1 Mm1
......................................................................ugagaucauugugUaaacugauu............... 5 1 Mm1
......................................................................ugagaucauCgugaaaacugauu............... 1 1 Mm1
......................................................................Ngagaucauugugaaaacugauu............... 7 1 Mm1
......................................................................ugagaucauugugaaaaAugauu............... 71 1 Mm1
......................................................................ugagaucUuugugaaaacugauu............... 2 1 Mm1
......................................................................ugagaucauugugaaaUcugauu............... 17 1 Mm1
......................................................................ugagauUauugugaaaacugauu............... 1 1 Mm1
......................................................................ugagaucauugugaaaacAgauu............... 2 1 Mm1
......................................................................ugagaucauuAugaaaacugauu............... 15 1 Mm1
......................................................................ugagaucaGugugaaaacugauu............... 2 1 Mm1
......................................................................ugagaucauugugaaaGcugauu............... 13 1 Mm1
......................................................................ugagaucauugugaaaacugauu............... 9341 0 Mm1
......................................................................ugagaucauugugaaaaUugauu............... 49 1 Mm1
......................................................................ugagaucGuugugaaaacugauu............... 33 1 Mm1
......................................................................ugagaucauugugaaaacugauuc.............. 2 0 Mm1
......................................................................ugagaucauugugaaaacugauuG.............. 3 1 Mm1
......................................................................ugagaucauugugaaaacugauuU.............. 813 1 Mm1
......................................................................ugagaucauugugaaaacugauuA.............. 41 1 Mm1
.......................................................................gagaucauAgugaaaacug.................. 1 1 Mm1
.......................................................................gagaucauugugaaaacuU.................. 1 1 Mm1
.......................................................................gagaucauugugaaaacug.................. 27 0 Mm1
.......................................................................gagaucauugugaaaacugau................ 1 0 Mm1
.......................................................................gagaucauugugaaaacugauu............... 2 0 Mm1
........................................................................Ugaucauugugaaaacug.................. 1 1 Mm1

...............................cugguuuucauaaugaccuucc....................................................... 1 0 Mm2
...............................cugguuuucauaaugaccuCcca...................................................... 1 1 Mm2
```

Star **Mature**

```
uguuggcuguaaugaugaccuucagacgaaacugguuuucauaaugaccuuccagauuguugugcuauucugagaucauugugaaaacugauuccguugcccccaauc
...............................cugguuuucauaaugacAuuccag.....................................................   1     1    Mm2
...............................cugguuuucauaaugaccuuccag.....................................................   3     0    Mm2
...............................cugguuuucauaaugaccuuccaga....................................................   29    0    Mm2
...............................cugguuuucauaaugaccuuccagU....................................................   3     1    Mm2
...............................cugguuuucauaaugaccuuGcaga....................................................   1     1    Mm2
...............................cugguuuucauaaugacUuuccaga....................................................   1     1    Mm2
...............................cugguuuucauaaugaUcuuccaga....................................................   1     1    Mm2
...................................uuuucauaaugaccuuccag.....................................................   1     0    Mm2
...................................uuuucauaaugaccuuccaga....................................................   10    0    Mm2
.....................................uucauaaugaccuuccaga....................................................   1     0    Mm2
...................................................................uucugagaucauugugCaaacu...................   2     1    Mm2
...................................................................uucugagaucauugugaGaacu...................   2     1    Mm2
...................................................................uucugagaucauugugaaaacG...................   1     1    Mm2
...................................................................uucugagaucauugugaaaacu...................   302   0    Mm2
...................................................................uucugagaucauugugaCaacu...................   1     1    Mm2
...................................................................uucAgagaucauugugaaaacu...................   1     1    Mm2
...................................................................uucugagaucauugugUaaacu...................   1     1    Mm2
...................................................................uucugagaucauugugaaaacuA..................   2     1    Mm2
...................................................................uucugagaucauugugaaaacuU..................   2     1    Mm2
...................................................................uucugagaucauugugaaaacug..................   12    0    Mm2
.....................................................................cugagaucauugugaaaacugau................   1     0    Mm2
.....................................................................cugagaucauugugaaaacugauu...............   3     0    Mm2
......................................................................ugagaucGuugugaaaac....................   2     1    Mm2
......................................................................ugagaucauugugaaaaA....................   36    1    Mm2
......................................................................ugagaucauugugaaaac....................   84    0    Mm2
......................................................................ugagaucauugugaaaaG....................   37    1    Mm2
......................................................................ugagaucauugugaaaaU....................   3     1    Mm2
......................................................................ugagaucauuCugaaaacu...................   2     1    Mm2
......................................................................ugagaucauugugaaGacu...................   1     1    Mm2
......................................................................ugagaucauugugaaaacG...................   20    1    Mm2
......................................................................ugagaucauugugaaaGcu...................   5     1    Mm2
......................................................................ugagaucauuUugaaaacu...................   3     1    Mm2
......................................................................ugagaucauugugaaaacu...................   2434  0    Mm2
......................................................................ugagaucauugugaaaUcu...................   5     1    Mm2
......................................................................ugagaCcauugugaaaacu...................   1     1    Mm2
......................................................................ugagaucauugAgaaaacu...................   2     1    Mm2
......................................................................ugagaucauugugaaaaAu...................   29    1    Mm2
......................................................................ugagaucUuugugaaaacu...................   1     1    Mm2
......................................................................Cgagaucauugugaaaacu...................   1     1    Mm2
......................................................................uAagaucauugugaaaacu...................   1     1    Mm2
......................................................................ugagaucauugugaaaaUu...................   15    1    Mm2
......................................................................Ngagaucauugugaaaacu...................   3     1    Mm2
......................................................................ugagauUauugugaaaacu...................   1     1    Mm2
......................................................................ugUgaucauugugaaaacu...................   1     1    Mm2
......................................................................ugagaucauugugaaaaGu...................   8     1    Mm2
......................................................................ugagaucauugugaaaCcu...................   2     1    Mm2
......................................................................ugagaucauugugaaaacA...................   1     1    Mm2
......................................................................ugagaucauCgugaaaacu...................   1     1    Mm2
......................................................................ugagaucGuugugaaaacu...................   7     1    Mm2
......................................................................ugagaucauugugaaaacuU..................   69    1    Mm2
......................................................................ugagaucauugugaaaGcug..................   30    1    Mm2
......................................................................ugagauNauugugaaaacug..................   1     1    Mm2
......................................................................uUagaucauugugaaaacug..................   7     1    Mm2
......................................................................ugagaucauuguCaaaacug..................   3     1    Mm2
......................................................................ugagGucauugugaaaacug..................   3     1    Mm2
......................................................................ugagaucauCgugaaaacug..................   3     1    Mm2
......................................................................ugagaucauuguAaaaacug..................   6     1    Mm2
......................................................................ugagaucauugugaaaaAug..................   224   1    Mm2
......................................................................ugagaucauugugGaaacug..................   2     1    Mm2
......................................................................ugagaucauugGgaaaacug..................   1     1    Mm2
......................................................................ugagaucauugugaaaacGg..................   156   1    Mm2
......................................................................ugagaucauugugaUaacug..................   1     1    Mm2
......................................................................ugagaucauugAgaaaacug..................   1     1    Mm2
......................................................................uAagaucauugugaaaacug..................   7     1    Mm2
......................................................................ugagaCcauugugaaaacug..................   3     1    Mm2
......................................................................ugagaucaCugugaaaacug..................   5     1    Mm2
......................................................................ugagaucaAugugaaaacug..................   1     1    Mm2
......................................................................ugagauUauugugaaaacug..................   9     1    Mm2
......................................................................ugagCucauugugaaaacug..................   2     1    Mm2
......................................................................ugagaucauugugaaGacug..................   1     1    Mm2
```

Star Mature

```
uguuggcuguaaugaugaccuucagacgaaacugguuuucauaaugaccuuccagauuguugugcuauucugagaucauugugaaaacugauuccguugcccccaauc
......................................................................ugGgaucauugugaaaacug..................   9       1   Mm2
......................................................................Ggagaucauugugaaaacug..................   1       1   Mm2
......................................................................ugagaAcauugugaaaacug..................   5       1   Mm2
......................................................................Ngagaucauugugaaaacug..................   12      1   Mm2
......................................................................ugagaucauuguUaaaacug..................   3       1   Mm2
......................................................................ugUgaucauugugaaaacug..................   3       1   Mm2
......................................................................ugagaucauuUugaaaacug..................   17      1   Mm2
......................................................................ugagaucauugugaaUacug..................   2       1   Mm2
......................................................................ugagaucUuugugaaaacug..................   5       1   Mm2
......................................................................ugagaucCuugugaaaacug..................   2       1   Mm2
......................................................................ugagaucauuAugaaaacug..................   57      1   Mm2
......................................................................ugagaucauugugaaaUcug..................   44      1   Mm2
......................................................................ugagaucauugugaaaaGug..................   119     1   Mm2
......................................................................ugaAaucauugugaaaacug..................   1       1   Mm2
......................................................................ugagaucGuugugaaaacug..................   10      1   Mm2
......................................................................ugagaucauugugaaaaUug..................   125     1   Mm2
......................................................................ugagaucauugugaaaacuA..................   106     1   Mm2
......................................................................ugagaucauugugaaaacug..................   27979   0   Mm2
......................................................................ugagaucauugugaaaCcug..................   53      1   Mm2
......................................................................ugagaucauugCgaaaacug..................   5       1   Mm2
......................................................................Cgagaucauugugaaaacug..................   7       1   Mm2
......................................................................ugCgaucauugugaaaacug..................   1       1   Mm2
......................................................................ugaCaucauugugaaaacug..................   1       1   Mm2
......................................................................ugagUucauugugaaaacug..................   3       1   Mm2
......................................................................ugagaNcauugugaaaacug..................   1       1   Mm2
......................................................................ugagaucauugugCaaacug..................   1       1   Mm2
......................................................................ugagaucauuCugaaaacug..................   22      1   Mm2
......................................................................Agagaucauugugaaaacug..................   4       1   Mm2
......................................................................ugaUaucauugugaaaacug..................   2       1   Mm2
......................................................................ugagaucauugugaaaacCg..................   4       1   Mm2
......................................................................ugagaucauugugaGaacug..................   2       1   Mm2
......................................................................ugagauAauugugaaaacug..................   2       1   Mm2
......................................................................ugagaucauugugUaaacug..................   6       1   Mm2
......................................................................ugagaucauugugaaaacuC..................   5       1   Mm2
......................................................................ugagaucauugugaCaacug..................   1       1   Mm2
......................................................................ugagUucauugugaaaacuga.................   9       1   Mm2
......................................................................ugagaucauugugaGaacuga.................   3       1   Mm2
......................................................................ugagaucaGugugaaaacuga.................   3       1   Mm2
......................................................................ugagaucauugugaaaacuUa.................   4       1   Mm2
......................................................................ugagaucauuCugaaaacuga.................   18      1   Mm2
......................................................................ugagaucauugugUaaacuga.................   3       1   Mm2
......................................................................Agagaucauugugaaaacuga.................   3       1   Mm2
......................................................................ugagaucauugugaaaacugC.................   9       1   Mm2
......................................................................ugaCaucauugugaaaacuga.................   1       1   Mm2
......................................................................ugagaucauugugaaaaUuga.................   112     1   Mm2
......................................................................ugagaucauuguUaaaacuga.................   2       1   Mm2
......................................................................ugagaGcauugugaaaacuga.................   1       1   Mm2
......................................................................ugagauGauugugaaaacuga.................   1       1   Mm2
......................................................................ugagaNcauugugaaaacuga.................   3       1   Mm2
......................................................................ugagaucauugugaaaUcuga.................   44      1   Mm2
......................................................................ugagaucauugugaaaCcuga.................   41      1   Mm2
......................................................................ugagaucauugugaaGacuga.................   3       1   Mm2
......................................................................ugagaucGuugugaaaacuga.................   23      1   Mm2
......................................................................ugagaucauugugaaaaGuga.................   131     1   Mm2
......................................................................ugGgaucauugugaaaacuga.................   4       1   Mm2
......................................................................ugagaucUuugugaaaacuga.................   4       1   Mm2
......................................................................ugUgaucauugugaaaacuga.................   1       1   Mm2
......................................................................ugagaucauCgugaaaacuga.................   2       1   Mm2
......................................................................ugagaucauugugGaaacuga.................   5       1   Mm2
......................................................................ugagaucauugCgaaaacuga.................   4       1   Mm2
......................................................................ugaUaucauugugaaaacuga.................   2       1   Mm2
......................................................................ugagaucauugAgaaaacuga.................   1       1   Mm2
......................................................................ugagaucauAgugaaaacuga.................   2       1   Mm2
......................................................................ugagaucauuUugaaaacuga.................   17      1   Mm2
......................................................................ugagaucauugugaaUacuga.................   4       1   Mm2
......................................................................ugagaucauuAugaaaacuga.................   47      1   Mm2
......................................................................ugagauNauugugaaaacuga.................   2       1   Mm2
......................................................................Ngagaucauugugaaaacuga.................   7       1   Mm2
......................................................................ugagaucauugugaaaaAuga.................   210     1   Mm2
......................................................................ugagaucauugugaaaacuAa.................   6       1   Mm2
```

```
                                               Star                            Mature
uguuggcuguaaugaugaccuucagacgaaacugguuuucauaaaugaccuuccagauuguugugcuauucugagaucauugugaaaacugauuccguugcccccaauc
.......................................................................ugagaucauugugCaaacuga.................   1       1    Mm2
.......................................................................ugagaucauugugaaaGcuga.................   28      1    Mm2
.......................................................................ugagaucauugugaaCacuga.................   1       1    Mm2
.......................................................................ugagCucauugugaaaacuga.................   2       1    Mm2
.......................................................................ugagauUauugugaaaacuga.................   8       1    Mm2
.......................................................................ugagGucauugugaaaacuga.................   3       1    Mm2
.......................................................................ugagaucCuugugaaaacuga.................   1       1    Mm2
.......................................................................ugagaAcauugugaaaacuga.................   4       1    Mm2
.......................................................................ugagaucauugugaaaacugU.................   393     1    Mm2
.......................................................................ugagaucauugugaaaacuga.................   24072   0    Mm2
.......................................................................uUagaucauugugaaaacuga.................   6       1    Mm2
.......................................................................ugagaucaAugugaaaacuga.................   2       1    Mm2
.......................................................................ugagaucaCugugaaaacuga.................   1       1    Mm2
.......................................................................ugagaCcauugugaaaacuga.................   3       1    Mm2
.......................................................................ugagaucauugugaaaacCga.................   2       1    Mm2
.......................................................................uAagaucauugugaaaacuga.................   9       1    Mm2
.......................................................................ugaAaucauugugaaaacuga.................   4       1    Mm2
.......................................................................Cgagaucauugugaaaacuga.................   7       1    Mm2
.......................................................................Ggagaucauugugaaaacuga.................   1       1    Mm2
.......................................................................ugagaucauugugaaaacugG.................   676     1    Mm2
.......................................................................ugagaucauugugaaUacugau................   1       1    Mm2
.......................................................................ugagaCcauugugaaaacugau................   1       1    Mm2
.......................................................................ugagaucauugugaaCacugau................   2       1    Mm2
.......................................................................ugagaucauugugaaaCcugau................   49      1    Mm2
.......................................................................ugagaucauugugaaaacugaA................   180     1    Mm2
.......................................................................ugagaucauugCgaaaacugau................   7       1    Mm2
.......................................................................ugagauGauugugaaaacugau................   1       1    Mm2
.......................................................................Cgagaucauugugaaaacugau................   9       1    Mm2
.......................................................................ugagaucauugugaUaacugau................   1       1    Mm2
.......................................................................ugagaucauuguCaaaacugau................   2       1    Mm2
.......................................................................ugUgaucauugugaaaacugau................   4       1    Mm2
.......................................................................ugagaucauugugaaaacCgau................   6       1    Mm2
.......................................................................ugagGucauugugaaaacugau................   4       1    Mm2
.......................................................................ugagaucauuAugaaaacugau................   59      1    Mm2
.......................................................................uCagaucauugugaaaacugau................   1       1    Mm2
.......................................................................ugagaGcauugugaaaacugau................   1       1    Mm2
.......................................................................ugagaucauuguUaaaacugau................   5       1    Mm2
.......................................................................ugagaucauugugaaaaAugau................   247     1    Mm2
.......................................................................ugagaucauugugaaaUcugau................   48      1    Mm2
.......................................................................Ngagaucauugugaaaacugau................   17      1    Mm2
.......................................................................ugagaucauugugaaaaUugau................   146     1    Mm2
.......................................................................ugagaucauugugaGaacugau................   1       1    Mm2
.......................................................................ugagaucauuUugaaaacugau................   16      1    Mm2
.......................................................................ugagaucaCugugaaaacugau................   4       1    Mm2
.......................................................................ugagaucauugugaaaacugUu................   6       1    Mm2
.......................................................................ugagaucauugugGaaacugau................   5       1    Mm2
.......................................................................ugagaucauugugaaaacugGu................   2       1    Mm2
.......................................................................ugaUaucauugugaaaacugau................   1       1    Mm2
.......................................................................ugagaucCuugugaaaacugau................   1       1    Mm2
.......................................................................ugagaucauGgugaaaacugau................   3       1    Mm2
.......................................................................ugagaucauugugaaGacugau................   2       1    Mm2
.......................................................................ugagaucauuguAaaaacugau................   3       1    Mm2
.......................................................................ugagaucauugugaaaacGgau................   2       1    Mm2
.......................................................................ugagaucauugugCaaacugau................   1       1    Mm2
.......................................................................ugagaucauugugaaaacugCu................   1       1    Mm2
.......................................................................ugGgaucauugugaaaacugau................   8       1    Mm2
.......................................................................ugagaucauugugaaaacugaG................   4       1    Mm2
.......................................................................ugagaucauugugaaaacuUau................   3       1    Mm2
.......................................................................ugagCucauugugaaaacugau................   2       1    Mm2
.......................................................................Agagaucauugugaaaacugau................   6       1    Mm2
.......................................................................ugagaucauugugaaaacugau................   27846   0    Mm2
.......................................................................uAagaucauugugaaaacugau................   13      1    Mm2
.......................................................................ugagauAauugugaaaacugau................   2       1    Mm2
.......................................................................ugagaucGuugugaaaacugau................   28      1    Mm2
.......................................................................ugagaNcauugugaaaacugau................   4       1    Mm2
.......................................................................ugagauUauugugaaaacugau................   6       1    Mm2
.......................................................................ugagaucauugAgaaaacugau................   2       1    Mm2
.......................................................................ugagaucauugugUaaacugau................   3       1    Mm2
.......................................................................ugagaucauugugaaaaGugau................   118     1    Mm2
.......................................................................ugaAaucauugugaaaacugau................   3       1    Mm2
```

Star **Mature**

uguuggcuguaaugaugaccuucagacgaaacugguuuucauaaugaccuuccagauuguugugcuauucugagaucauugugaaaacugauuccguugcccccaauc			
....ugagaucauugugaaaacuAau................	1	1	Mm2
....ugagaucauCgugaaaacugau................	3	1	Mm2
....ugagaucauugugaaaGcugau................	35	1	Mm2
....ugagaucauugugaaaacAgau................	2	1	Mm2
....ugagaucauugugaaaacugaC................	8	1	Mm2
....ugagaucauAgugaaaacugau................	3	1	Mm2
....ugagaucUuugugaaaacugau................	8	1	Mm2
....ugagaAcauugugaaaacugau................	1	1	Mm2
....uUagaucauugugaaaacugau................	10	1	Mm2
....ugagaucauuCugaaaacugau................	36	1	Mm2
....ugagaucauugAgaaaacugauu...............	8	1	Mm2
....ugagaucauuguCaaaacugauu...............	4	1	Mm2
....ugagaucauugugaaaacugauC...............	7	1	Mm2
....ugagaucauugugaaaacugGuu...............	6	1	Mm2
....uUagaucauugugaaaacugauu...............	11	1	Mm2
....ugagaucauugugaGaacugauu...............	3	1	Mm2
....ugagaucaCugugaaaacugauu...............	3	1	Mm2
....ugagaucaAugugaaaacugauu...............	5	1	Mm2
....ugagaucauugugaaaacugauu...............	34937	0	Mm2
....ugagaucauugugaaaacCgauu...............	6	1	Mm2
....ugagGucauugugaaaacugauu...............	3	1	Mm2
....ugagaucauugugaaaacugaCu...............	2	1	Mm2
....ugagaucaGugugaaaacugauu...............	2	1	Mm2
....ugagaNcauugugaaaacugauu...............	8	1	Mm2
....ugagaucauugugGaaacugauu...............	3	1	Mm2
....ugagaucauugugaaaacuUauu...............	7	1	Mm2
....ugagauAauugugaaaacugauu...............	2	1	Mm2
....ugagCucauugugaaaacugauu...............	13	1	Mm2
....ugagaucauugugaaaacugauA...............	361	1	Mm2
....ugagaucauugugaCaacugauu...............	1	1	Mm2
....ugUgaucauugugaaaacugauu...............	4	1	Mm2
....ugagaucauugugaaaacugaGu...............	1	1	Mm2
....ugagaucauuCugaaaacugauu...............	29	1	Mm2
....ugagaucauugugaaaacAgauu...............	3	1	Mm2
....ugagaucauugugaaaacugCuu...............	1	1	Mm2
....ugagaucauugCgaaaacugauu...............	9	1	Mm2
....ugaCaucauugugaaaacugauu...............	1	1	Mm2
....ugagaucauugugaaaacugUuu...............	16	1	Mm2
....ugagaucauugugaaaaGugauu...............	140	1	Mm2
....uAagaucauugugaaaacugauu...............	22	1	Mm2
....ugagaucauugugaaaacugauG...............	9	1	Mm2
....ugagaucUuugugaaaacugauu...............	4	1	Mm2
....ugagaucauuguAaaaacugauu...............	4	1	Mm2
....ugagaucauugugaaaacGgauu...............	1	1	Mm2
....ugagaucGuugugaaaacugauu...............	90	1	Mm2
....ugagaAcauugugaaaacugauu...............	1	1	Mm2
....Ngagaucauugugaaaacugauu...............	18	1	Mm2
....ugagauUauugugaaaacugauu...............	11	1	Mm2
....ugCgaucauugugaaaacugauu...............	5	1	Mm2
....ugagaucauAgugaaaacugauu...............	2	1	Mm2
....ugagaucauugugaaaCcugauu...............	69	1	Mm2
....ugagauNauugugaaaacugauu...............	2	1	Mm2
....Cgagaucauugugaaaacugauu...............	10	1	Mm2
....ugagaucauugugUaaacugauu...............	5	1	Mm2
....ugagaucauugugaaaaAugauu...............	282	1	Mm2
....ugGgaucauugugaaaacugauu...............	14	1	Mm2
....ugagaucauugugaaCacugauu...............	1	1	Mm2
....ugagaucauugugaaaGcugauu...............	38	1	Mm2
....ugagaucauuUugaaaacugauu...............	23	1	Mm2
....Ggagaucauugugaaaacugauu...............	1	1	Mm2
....ugagaucauugugaaaaUugauu...............	173	1	Mm2
....ugagaucauugugaaaacugaAu...............	12	1	Mm2
....ugagaCcauugugaaaacugauu...............	2	1	Mm2
....ugagaucauugugCaaacugauu...............	3	1	Mm2
....ugaUaucauugugaaaacugauu...............	2	1	Mm2
....ugagaucauugugaaGacugauu...............	5	1	Mm2
....Agagaucauugugaaaacugauu...............	6	1	Mm2
....ugagaucauugugaaUacugauu...............	5	1	Mm2
....ugaAaucauugugaaaacugauu...............	2	1	Mm2
....uCagaucauugugaaaacugauu...............	4	1	Mm2

Star **Mature**

```
uguuggcuguaaugaugaccuucagacgaaacugguuuucauaaugaccuuccagauuguugugcuauucugagaucauugugaaaacugauuccguugcccccaauc
......................................................................ugagaucauCgugaaaacugauu...............   3     1   Mm2
......................................................................ugagaucauuAugaaaacugauu...............   60    1   Mm2
......................................................................ugagaucauGgugaaaacugauu...............   4     1   Mm2
......................................................................ugagaucauugugaaaUcugauu...............   70    1   Mm2
......................................................................ugagaucauugugaUaacugauu...............   4     1   Mm2
......................................................................uNagaucauugugaaaacugauu...............   1     1   Mm2
......................................................................ugagaucCuugugaaaacugauu...............   3     1   Mm2
......................................................................ugagUucauugugaaaacugauu...............   8     1   Mm2
......................................................................ugagaucauugugaaaacugauuA..............   126   1   Mm2
......................................................................ugagaucauugugaaaacugauuG..............   4     1   Mm2
......................................................................ugagaucauugugaaaacugauuU..............   2922  1   Mm2
......................................................................ugagaucauugugaaaaGugauuc..............   1     1   Mm2
......................................................................ugagaucauugugaaaacugauuc..............   5     0   Mm2
......................................................................ugagaucauugugaaaacugauucU.............   1     1   Mm2
.......................................................................gGgaucauugugaaaacu...................   1     1   Mm2
.......................................................................gagaucauugugaaaacug..................   14    0   Mm2
.......................................................................Aagaucauugugaaaacuga.................   1     1   Mm2
.......................................................................gagaucauugugaaaacuga.................   2     0   Mm2
.......................................................................Uagaucauugugaaaacugau................   1     1   Mm2
.......................................................................gagaucauugugaaaacugau................   5     0   Mm2
.......................................................................Uagaucauugugaaaacugauu...............   1     1   Mm2
.......................................................................gagaucauugugaaaacugauu...............   4     0   Mm2
........................................................................Ugaucauugugaaaacug..................   1     1   Mm2
........................................................................Ugaucauugugaaaacugauu...............   1     1   Mm2

...............................cugguuuucauaaugaccuucc.......................................................   5     0   Fm1
...............................cugguuuucauaaugaccuucG.......................................................   1     1   Fm1
...............................cugguuuucauaaugaccuucca......................................................   3     0   Fm1
...............................cugguuuucauaaugaccuuccG......................................................   1     1   Fm1
...............................cugguuuucauaaugacUuuccag.....................................................   2     1   Fm1
...............................cugguuuucaGaaugaccuuccag.....................................................   1     1   Fm1
...............................cugguuuucauaaugaccuuccag.....................................................   35    0   Fm1
...............................cugguuuucauaaugaccuuccagU....................................................   4     1   Fm1
...............................cugguuuucauaaugacUuuccaga....................................................   1     1   Fm1
...............................cugguNuucauaaugaccuuccaga....................................................   1     1   Fm1
...............................cugguuuucauaaugacAuuccaga....................................................   1     1   Fm1
...............................cugguuuucaCaaugaccuuccaga....................................................   2     1   Fm1
...............................cugguuuucauaaugaccuuccaga....................................................   119   0   Fm1
.................................gguuuucauaaugaccuuccagU....................................................   4     1   Fm1
...................................uuuucauaaugaccuuccaga....................................................   5     0   Fm1
...................................................................uucugagaucauugugaaa......................   6     0   Fm1
...................................................................uucugagaucauugugaaaa.....................   3     0   Fm1
...................................................................uucugagaucauugugaaaac....................   10    0   Fm1
...................................................................uucugagaucauugugaaaaU....................   5     1   Fm1
...................................................................uucugagaucauugugaUaacu...................   1     1   Fm1
...................................................................uucugGgaucauugugaaaacu...................   1     1   Fm1
...................................................................uucugagaucCuugugaaaacu...................   1     1   Fm1
...................................................................uuAugagaucauugugaaaacu...................   1     1   Fm1
...................................................................uucugagaucauugugaGaacu...................   2     1   Fm1
...................................................................uucugagaucauugugaaaacu...................   636   0   Fm1
...................................................................uAcugagaucauugugaaaacu...................   1     1   Fm1
...................................................................uucuAagaucauugugaaaacu...................   2     1   Fm1
...................................................................uuGugagaucauugugaaaacu...................   1     1   Fm1
...................................................................Cucugagaucauugugaaaacu...................   2     1   Fm1
...................................................................uucugagaucUuugugaaaacu...................   2     1   Fm1
...................................................................uucugagaucauugugaaaacG...................   3     1   Fm1
...................................................................uucugagaucGuugugaaaacu...................   6     1   Fm1
...................................................................uucugagaucauugugCaaacu...................   2     1   Fm1
...................................................................uucugagaucauugugUaaacu...................   1     1   Fm1
...................................................................uucugagaucauugGgaaaacu...................   1     1   Fm1
...................................................................uucugagaucauugugaaaaUu...................   1     1   Fm1
...................................................................uucugagaucauugugaaaacug..................   32    0   Fm1
...................................................................uucugagaucauugugaaaacuU..................   6     1   Fm1
...................................................................uucugagaucauugugGaaacug..................   1     1   Fm1
...................................................................uucugagaucauugugaUaacug..................   1     1   Fm1
...................................................................uucugagaucauugugaaaacuA..................   8     1   Fm1
....................................................................ucugagaucauugugaaaacug..................   7     0   Fm1
.....................................................................cugagaucauugugaaaacGg..................   1     1   Fm1
.....................................................................cugagaucauugugaaaacug..................   5     0   Fm1
.....................................................................cugagaucauugugaaaacugauu...............   6     0   Fm1
```

```
                                   Star                              Mature
uguuggcuguaaugaugaccuucagacgaaacugguuuucauaaugaccuuccagauuguugugcuauucugagaucauugugaaaacugauuccguugcccccaauc
......................................................................ugagaucGuugugaaaac....................  3       1   Fm1
......................................................................ugagaucauugugaaaGc....................  1       1   Fm1
......................................................................ugagauNauugugaaaac....................  1       1   Fm1
......................................................................ugagaucauugugaaaac....................  186     0   Fm1
......................................................................ugagaucauugugaaaaU....................  24      1   Fm1
......................................................................ugagaucauugugaaaaG....................  106     1   Fm1
......................................................................ugagaucauuCugaaaac....................  1       1   Fm1
......................................................................ugagaucauugugaaaaA....................  133     1   Fm1
......................................................................ugagaucUuugugaaaacu...................  2       1   Fm1
......................................................................ugagaAcauugugaaaacu...................  5       1   Fm1
......................................................................ugCgaucauugugaaaacu...................  2       1   Fm1
......................................................................ugagaucauugugaaaacu...................  10860   0   Fm1
......................................................................ugagaucauAgugaaaacu...................  1       1   Fm1
......................................................................ugagaucauugugaaaaUu...................  65      1   Fm1
......................................................................ugagaucauugugaaaacG...................  117     1   Fm1
......................................................................ugagaucauugugaGaacu...................  11      1   Fm1
......................................................................ugagaucauuguUaaaacu...................  2       1   Fm1
......................................................................ugagaucauugugaUaacu...................  1       1   Fm1
......................................................................ugGgaucauugugaaaacu...................  3       1   Fm1
......................................................................Ngagaucauugugaaaacu...................  4       1   Fm1
......................................................................ugagaucauCgugaaaacu...................  1       1   Fm1
......................................................................ugagaucauuCugaaaacu...................  10      1   Fm1
......................................................................ugagaucauugugaaaaGu...................  52      1   Fm1
......................................................................ugagauUauugugaaaacu...................  6       1   Fm1
......................................................................ugagaCcauugugaaaacu...................  1       1   Fm1
......................................................................ugagaucauugAgaaaacu...................  3       1   Fm1
......................................................................uAagaucauugugaaaacu...................  7       1   Fm1
......................................................................ugagaucauugCgaaaacu...................  1       1   Fm1
......................................................................ugagaucGuugugaaaacu...................  45      1   Fm1
......................................................................ugagaucaCugugaaaacu...................  1       1   Fm1
......................................................................uUagaucauugugaaaacu...................  1       1   Fm1
......................................................................ugagaNcauugugaaaacu...................  3       1   Fm1
......................................................................ugagaucauugugaaaacC...................  3       1   Fm1
......................................................................ugagUucauugugaaaacu...................  1       1   Fm1
......................................................................ugagaucauugugaaaacA...................  24      1   Fm1
......................................................................ugagCucauugugaaaacu...................  3       1   Fm1
......................................................................ugagaucauugugaaaGcu...................  10      1   Fm1
......................................................................ugagaucauuUugaaaacu...................  6       1   Fm1
......................................................................ugaCaucauugugaaaacu...................  2       1   Fm1
......................................................................ugagaucCuugugaaaacu...................  2       1   Fm1
......................................................................ugagaucauugugaaaUcu...................  25      1   Fm1
......................................................................ugagGucauugugaaaacu...................  4       1   Fm1
......................................................................ugagauAauugugaaaacu...................  2       1   Fm1
......................................................................ugagaucaAugugaaaacu...................  2       1   Fm1
......................................................................ugUgaucauugugaaaacu...................  5       1   Fm1
......................................................................Cgagaucauugugaaaacu...................  3       1   Fm1
......................................................................ugagauNauugugaaaacu...................  2       1   Fm1
......................................................................ugagaucauugugaaaaAu...................  114     1   Fm1
......................................................................ugagaucauugugaaGacu...................  3       1   Fm1
......................................................................ugagaucauuguAaaaacu...................  3       1   Fm1
......................................................................ugagaucauugugaaaCcu...................  14      1   Fm1
......................................................................ugagaucauugugGaaacu...................  1       1   Fm1
......................................................................ugagaNcauugugaaaacug..................  30      1   Fm1
......................................................................ugagCucauugugaaaacug..................  22      1   Fm1
......................................................................ugagaucauugugaaUacug..................  17      1   Fm1
......................................................................ugagaucauugugaaaacug..................  195850  0   Fm1
......................................................................ugagaucauugugaaaacuC..................  4       1   Fm1
......................................................................ugagaucauugugaaaacGg..................  1343    1   Fm1
......................................................................ugUgaucauugugaaaacug..................  37      1   Fm1
......................................................................ugagaucauugugCaaacug..................  14      1   Fm1
......................................................................ugagaucauuguAaaaacug..................  27      1   Fm1
......................................................................ugagaucauugugaGaacug..................  15      1   Fm1
......................................................................ugagaucauugugaaaacuU..................  321     1   Fm1
......................................................................Cgagaucauugugaaaacug..................  64      1   Fm1
......................................................................ugagauAauugugaaaacug..................  10      1   Fm1
......................................................................uAagaucauugugaaaacug..................  62      1   Fm1
......................................................................ugagaAcauugugaaaacug..................  17      1   Fm1
......................................................................uNagaucauugugaaaacug..................  1       1   Fm1
......................................................................ugagaucauugugaaGacug..................  40      1   Fm1
......................................................................ugagauGauugugaaaacug..................  4       1   Fm1
```

Star Mature

```
uguuggcuguaaugaugaccuucagacgaaacugguuuucauaaugaccuuccagauuguugugcuauucugagaucauugugaaaacugauuccguugcccccaauc
......................................................................Ngagaucauugugaaaacug.................. 94 1 Fm1
......................................................................ugagaucauugugaaaaNug.................. 1 1 Fm1
......................................................................ugagaucCuugugaaaacug.................. 3 1 Fm1
......................................................................ugagaucauCgugaaaacug.................. 29 1 Fm1
......................................................................ugagaucauuguUaaaacug.................. 21 1 Fm1
......................................................................ugagaucauugugaaCacug.................. 4 1 Fm1
......................................................................ugagaCcauugugaaaacug.................. 8 1 Fm1
......................................................................ugagaucaCugugaaaacug.................. 13 1 Fm1
......................................................................ugGgaucauugugaaaacug.................. 69 1 Fm1
......................................................................ugagGucauugugaaaacug.................. 27 1 Fm1
......................................................................ugagaucauugugaaaacuA.................. 399 1 Fm1
......................................................................ugagaucauugAgaaaacug.................. 15 1 Fm1
......................................................................ugagaucaAugugaaaacug.................. 6 1 Fm1
......................................................................ugagaucauugugaaaUcug.................. 376 1 Fm1
......................................................................ugagaucauGgugaaaacug.................. 10 1 Fm1
......................................................................ugagaucauugGgaaaacug.................. 6 1 Fm1
......................................................................ugaCaucauugugaaaacug.................. 10 1 Fm1
......................................................................ugagaucauugugaaaacAg.................. 12 1 Fm1
......................................................................ugagaucauuguCaaaacug.................. 9 1 Fm1
......................................................................uCagaucauugugaaaacug.................. 16 1 Fm1
......................................................................uUagaucauugugaaaacug.................. 27 1 Fm1
......................................................................ugagaucauugugaaaaAug.................. 1638 1 Fm1
......................................................................ugagaucauugugaaaGcug.................. 220 1 Fm1
......................................................................Ggagaucauugugaaaacug.................. 8 1 Fm1
......................................................................Agagaucauugugaaaacug.................. 24 1 Fm1
......................................................................ugagaucauugugUaaacug.................. 36 1 Fm1
......................................................................ugagaucauugugaaaaGug.................. 868 1 Fm1
......................................................................ugagaucUuugugaaaacug.................. 40 1 Fm1
......................................................................ugagaucGuugugaaaacug.................. 44 1 Fm1
......................................................................ugagaucauugCgaaaacug.................. 30 1 Fm1
......................................................................ugagaucaGugugaaaacug.................. 13 1 Fm1
......................................................................ugCgaucauugugaaaacug.................. 20 1 Fm1
......................................................................ugagaucauuAugaaaacug.................. 418 1 Fm1
......................................................................ugagaucauAgugaaaacug.................. 12 1 Fm1
......................................................................ugagaucauugugaCaacug.................. 5 1 Fm1
......................................................................ugagaucauugugaUaacug.................. 12 1 Fm1
......................................................................ugagaucauugugaaaaUug.................. 979 1 Fm1
......................................................................ugaUaucauugugaaaacug.................. 13 1 Fm1
......................................................................ugagaucauuUugaaaacug.................. 137 1 Fm1
......................................................................ugagauNauugugaaaacug.................. 18 1 Fm1
......................................................................ugNgaucauugugaaaacug.................. 1 1 Fm1
......................................................................ugagUucauugugaaaacug.................. 25 1 Fm1
......................................................................ugagauUauugugaaaacug.................. 48 1 Fm1
......................................................................ugagaGcauugugaaaacug.................. 8 1 Fm1
......................................................................ugaAaucauugugaaaacug.................. 30 1 Fm1
......................................................................ugagaucauugugaaaacCg.................. 31 1 Fm1
......................................................................ugagaucauugugGaaacug.................. 36 1 Fm1
......................................................................ugagaucauugugaaaCcug.................. 375 1 Fm1
......................................................................ugagaucauuCugaaaacug.................. 181 1 Fm1
......................................................................ugagaucauugugaaaacuga................. 89737 0 Fm1
......................................................................ugagaucaAugugaaaacuga................. 7 1 Fm1
......................................................................ugaUaucauugugaaaacuga................. 5 1 Fm1
......................................................................ugagaucauugugaaUacuga................. 7 1 Fm1
......................................................................ugagaucauugugaaaacuAa................. 11 1 Fm1
......................................................................ugagaucauugugaaaUcuga................. 199 1 Fm1
......................................................................uUagaucauugugaaaacuga................. 18 1 Fm1
......................................................................ugagaCcauugugaaaacuga................. 6 1 Fm1
......................................................................ugagUucauugugaaaacuga................. 8 1 Fm1
......................................................................ugagaucauugugaaCacuga................. 2 1 Fm1
......................................................................Agagaucauugugaaaacuga................. 4 1 Fm1
......................................................................ugGgaucauugugaaaacuga................. 31 1 Fm1
......................................................................ugagaucauugugaaaacCga................. 9 1 Fm1
......................................................................ugagaucauCgugaaaacuga................. 20 1 Fm1
......................................................................ugagaucauugugaCaacuga................. 2 1 Fm1
......................................................................ugagGucauugugaaaacuga................. 14 1 Fm1
......................................................................ugagaucauugugaaaaAuga................. 753 1 Fm1
......................................................................ugagaucaGugugaaaacuga................. 5 1 Fm1
......................................................................ugagaucauugugaaaacugU................. 1923 1 Fm1
......................................................................ugagaucauugugUaaacuga................. 16 1 Fm1
......................................................................ugagaucauugCgaaaacuga................. 13 1 Fm1
```

```
                                   Star                                Mature
uguuggcuguaaugaugaccuucagacgaaacugguuuucauaaugaccuuccagauuguugugcuauucugagaucauugugaaaacugauuccguugcccccaauc
......................................................................ugagaucauugugaaaacuCa.................	3	1	Fm1
......................................................................Cgagaucauugugaaaacuga.................	17	1	Fm1
......................................................................ugagaucauugugaaaacugC.................	53	1	Fm1
......................................................................ugagaucauugugGaaacuga.................	21	1	Fm1
......................................................................ugagaucauugugaaaGcuga.................	110	1	Fm1
......................................................................ugagaucGuugugaaaacuga.................	77	1	Fm1
......................................................................uNagaucauugugaaaacuga.................	2	1	Fm1
......................................................................ugagaucauugugaaaNcuga.................	1	1	Fm1
......................................................................ugagaucauugugaaGacuga.................	10	1	Fm1
......................................................................ugagaucUuugugaaaacuga.................	20	1	Fm1
......................................................................ugagaucauGgugaaaacuga.................	2	1	Fm1
......................................................................ugagaucauugugaaaacAga.................	3	1	Fm1
......................................................................ugagaucauugGgaaaacuga.................	2	1	Fm1
......................................................................ugagauAauugugaaaacuga.................	6	1	Fm1
......................................................................ugagaucauugugaaaacugG.................	4306	1	Fm1
......................................................................ugagaucauugAgaaaacuga.................	3	1	Fm1
......................................................................ugagaucauuguCaaaacuga.................	5	1	Fm1
......................................................................ugagaucauuCugaaaacuga.................	97	1	Fm1
......................................................................ugagaucaCugugaaaacuga.................	5	1	Fm1
......................................................................ugagCucauugugaaaacuga.................	15	1	Fm1
......................................................................ugagaNcauugugaaaacuga.................	17	1	Fm1
......................................................................ugagaGcauugugaaaacuga.................	2	1	Fm1
......................................................................ugagaucauuUugaaaacuga.................	72	1	Fm1
......................................................................ugagaucauuguAaaaacuga.................	12	1	Fm1
......................................................................ugagaucauugugaaaCcuga.................	130	1	Fm1
......................................................................ugUgaucauugugaaaacuga.................	14	1	Fm1
......................................................................ugagauGauugugaaaacuga.................	4	1	Fm1
......................................................................ugagauNauugugaaaacuga.................	2	1	Fm1
......................................................................ugagaucauugugaUaacuga.................	4	1	Fm1
......................................................................ugCgaucauugugaaaacuga.................	8	1	Fm1
......................................................................ugagaucauugugCaaacuga.................	4	1	Fm1
......................................................................ugagaucCuugugaaaacuga.................	3	1	Fm1
......................................................................ugaCaucauugugaaaacuga.................	5	1	Fm1
......................................................................ugagaucauugugaaaaUuga.................	450	1	Fm1
......................................................................uAagaucauugugaaaacuga.................	33	1	Fm1
......................................................................ugagaucauugugaaaacuUa.................	13	1	Fm1
......................................................................ugagaucauuAugaaaacuga.................	167	1	Fm1
......................................................................ugagaucauAgugaaaacuga.................	4	1	Fm1
......................................................................uCagaucauugugaaaacuga.................	6	1	Fm1
......................................................................ugagauUauugugaaaacuga.................	29	1	Fm1
......................................................................ugagaAcauugugaaaacuga.................	7	1	Fm1
......................................................................ugNgaucauugugaaaacuga.................	1	1	Fm1
......................................................................ugagaucauugugaaaaGuga.................	383	1	Fm1
......................................................................Ngagaucauugugaaaacuga.................	45	1	Fm1
......................................................................ugagaucauugugaGaacuga.................	10	1	Fm1
......................................................................ugaAaucauugugaaaacuga.................	11	1	Fm1
......................................................................ugagaucauuguUaaaacuga.................	9	1	Fm1
......................................................................ugagaucauugugaGaacugau................	13	1	Fm1
......................................................................ugCgaucauugugaaaacugau................	12	1	Fm1
......................................................................ugaAaucauugugaaaacugau................	10	1	Fm1
......................................................................ugagUucauugugaaaacugau................	19	1	Fm1
......................................................................ugGgaucauugugaaaacugau................	36	1	Fm1
......................................................................ugagaucauAgugaaaacugau................	9	1	Fm1
......................................................................ugagauGauugugaaaacugau................	4	1	Fm1
......................................................................ugagaucauugugaaaUcugau................	170	1	Fm1
......................................................................Ggagaucauugugaaaacugau................	1	1	Fm1
......................................................................ugagaNcauugugaaaacugau................	13	1	Fm1
......................................................................ugagaucauugugaaaacAgau................	3	1	Fm1
......................................................................ugagGucauugugaaaacugau................	10	1	Fm1
......................................................................ugaCaucauugugaaaacugau................	3	1	Fm1
......................................................................ugagaucauuUugaaaacugau................	74	1	Fm1
......................................................................ugagaucauugugaaaaUugau................	522	1	Fm1
......................................................................ugagaucauugugaaUacugau................	8	1	Fm1
......................................................................Cgagaucauugugaaaacugau................	40	1	Fm1
......................................................................ugagaucauuguAaaaacugau................	9	1	Fm1
......................................................................ugagCucauugugaaaacugau................	28	1	Fm1
......................................................................ugagaucauugugaaaacuAau................	7	1	Fm1
......................................................................ugagaucauugugaaaacGgau................	1	1	Fm1
......................................................................ugagaucauugugaaGacugau................	18	1	Fm1
......................................................................Agagaucauugugaaaacugau................	8	1	Fm1
```

```
                                                    Star                              Mature
uguuggcuguaaugaugaccuucagacgaaacugguuuucauaaugaccuuccagauuguugugcuauucugagaucauugugaaaacugauuccguugcccccaauc
......................................................................ugagaucauuguCaaaacugau................   5       1   Fm1
......................................................................ugagaucauugugUaaacugau................   21      1   Fm1
......................................................................ugagaAcauugugaaaacugau................   5       1   Fm1
......................................................................ugagaucauugugaaaacugCu................   11      1   Fm1
......................................................................ugagaucauugugaaaacuUau................   11      1   Fm1
......................................................................Ngagaucauugugaaaacugau................   51      1   Fm1
......................................................................ugagaucUuugugaaaacugau................   25      1   Fm1
......................................................................ugagaucaAugugaaaacugau................   3       1   Fm1
......................................................................ugagaucauugugGaaacugau................   17      1   Fm1
......................................................................ugagaCcauugugaaaacugau................   4       1   Fm1
......................................................................ugagaucauugugaaaaCCgau................   21      1   Fm1
......................................................................ugagaucauugugaaaacuCau................   3       1   Fm1
......................................................................ugagaucaCugugaaaacugau................   7       1   Fm1
......................................................................ugagaucauuCugaaaacugau................   93      1   Fm1
......................................................................ugagaucauugugaaaacugaG................   15      1   Fm1
......................................................................ugagaucGuugugaaaacugau................   75      1   Fm1
......................................................................ugagaucauCgugaaaacugau................   18      1   Fm1
......................................................................ugagaucauugAgaaaacugau................   10      1   Fm1
......................................................................uAagaucauugugaaaacugau................   41      1   Fm1
......................................................................ugagaucauugugaaaacugaA................   760     1   Fm1
......................................................................ugagauNauugugaaaacugau................   2       1   Fm1
......................................................................ugagaucauuAugaaaacugau................   211     1   Fm1
......................................................................uUagaucauugugaaaacugau................   14      1   Fm1
......................................................................ugagaucauugugaaCacugau................   1       1   Fm1
......................................................................ugagaucauugGgaaaacugau................   3       1   Fm1
......................................................................ugagaucauugugaaaGcugau................   157     1   Fm1
......................................................................ugaUaucauugugaaaacugau................   4       1   Fm1
......................................................................ugagaucauGgugaaaacugau................   4       1   Fm1
......................................................................ugagaucauugugaaaaAugau................   923     1   Fm1
......................................................................ugagaucauugugaaaacugUu................   54      1   Fm1
......................................................................ugagaucauuguUaaaacugau................   19      1   Fm1
......................................................................uCagaucauugugaaaacugau................   7       1   Fm1
......................................................................ugagaucauugugaCaacugau................   2       1   Fm1
......................................................................ugagaucauugugaaaCcugau................   166     1   Fm1
......................................................................ugagaucauugugaUaacugau................   6       1   Fm1
......................................................................ugagaGcauugugaaaacugau................   1       1   Fm1
......................................................................ugUgaucauugugaaaacugau................   17      1   Fm1
......................................................................uNagaucauugugaaaacugau................   3       1   Fm1
......................................................................ugagaucauugugaaaacugaC................   30      1   Fm1
......................................................................ugagaucauugugCaaacugau................   9       1   Fm1
......................................................................ugagaucCuugugaaaacugau................   2       1   Fm1
......................................................................ugagauAauugugaaaacugau................   9       1   Fm1
......................................................................ugagauUauugugaaaacugau................   44      1   Fm1
......................................................................ugagaucauugCgaaaacugau................   29      1   Fm1
......................................................................ugagaucauugugaaaacugGu................   17      1   Fm1
......................................................................ugagaucauugugaaaacugau................   105108  0   Fm1
......................................................................ugagaucauugugaaaaGugau................   446     1   Fm1
......................................................................ugagaucauugCgaaaacugauu...............   32      1   Fm1
......................................................................uAagaucauugugaaaacugauu...............   61      1   Fm1
......................................................................ugagaucauuguUaaaacugauu...............   24      1   Fm1
......................................................................ugagaucauugugaaaaUugauu...............   909     1   Fm1
......................................................................ugagaucauugugUaaacugauu...............   30      1   Fm1
......................................................................ugagaucauugAgaaaacugauu...............   24      1   Fm1
......................................................................ugagaucaGugugaaaacugauu...............   7       1   Fm1
......................................................................ugagaucauugugaGaacugauu...............   33      1   Fm1
......................................................................ugagaucaCugugaaaacugauu...............   22      1   Fm1
......................................................................ugagauGauugugaaaacugauu...............   9       1   Fm1
......................................................................ugagaucauugugaaaaAugauu...............   1579    1   Fm1
......................................................................ugagaucauugugaaaaNugauu...............   1       1   Fm1
......................................................................ugagaucauugugGaaacugauu...............   42      1   Fm1
......................................................................ugagaucGuugugaaaacugauu...............   346     1   Fm1
......................................................................ugagaucauugugaaaacugauA...............   1483    1   Fm1
......................................................................ugagaucauugugaaUacugauu...............   28      1   Fm1
......................................................................uUagaucauugugaaaacugauu...............   23      1   Fm1
......................................................................ugagaucauugugaaaacuUauu...............   13      1   Fm1
......................................................................ugagaucauugugaaaaGugauu...............   811     1   Fm1
......................................................................ugagaucauugugaaaacuAauu...............   21      1   Fm1
......................................................................ugagaucauugugaaaacugaCu...............   15      1   Fm1
......................................................................ugagaucauugugaaaacugUuu...............   59      1   Fm1
......................................................................ugagaCcauugugaaaacugauu...............   23      1   Fm1
```

Star / Mature

```
uguuggcuguaaugaugaccuucagacgaaacugguuuucauaaugaccuuccagauuguugugcuauucugagaucauugugaaaacugauuccguugcccccaauc
......................................................................ugagaucauGgugaaaacugauu...............    13      1    Fm1
......................................................................ugagGucauugugaaaacugauu...............    23      1    Fm1
......................................................................uCagaucauugugaaaacugauu...............    15      1    Fm1
......................................................................ugaCaucauugugaaaacugauu...............    10      1    Fm1
......................................................................ugagaucauuAugaaaacugauu...............    411     1    Fm1
......................................................................Ggagaucauugugaaaacugauu...............    8       1    Fm1
......................................................................ugagaucauugugaaaacugGuu...............    24      1    Fm1
......................................................................ugagaucauugugaaaacugauG...............    24      1    Fm1
......................................................................ugaAaucauugugaaaacugauu...............    18      1    Fm1
......................................................................ugagaucauugugaaaacuCauu...............    7       1    Fm1
......................................................................ugNgaucauugugaaaacugauu...............    1       1    Fm1
......................................................................ugagaucauugugaaaGcugauu...............    224     1    Fm1
......................................................................ugagaucauuUugaaaacugauu...............    127     1    Fm1
......................................................................ugagaucauugugaCaacugauu...............    4       1    Fm1
......................................................................ugagauNauugugaaaacugauu...............    11      1    Fm1
......................................................................ugagCucauugugaaaacugauu...............    45      1    Fm1
......................................................................ugaUaucauugugaaaacugauu...............    9       1    Fm1
......................................................................ugagaucUuugugaaaacugauu...............    37      1    Fm1
......................................................................Cgagaucauugugaaaacugauu...............    65      1    Fm1
......................................................................ugagaucauugugaaaacCgauu...............    18      1    Fm1
......................................................................ugagaucauugugaaaacugaAu...............    47      1    Fm1
......................................................................ugagaucaAugugaaaacugauu...............    13      1    Fm1
......................................................................ugagaucauCgugaaaacugauu...............    30      1    Fm1
......................................................................ugagaucauugugCaaacugauu...............    10      1    Fm1
......................................................................ugagUucauugugaaaacugauu...............    35      1    Fm1
......................................................................ugagaucauugugaaaUcugauu...............    322     1    Fm1
......................................................................ugagauUauugugaaaacugauu...............    71      1    Fm1
......................................................................ugagaucauugugaaaCcugauu...............    318     1    Fm1
......................................................................ugagaucauugugaUaacugauu...............    20      1    Fm1
......................................................................ugagauAauugugaaaacugauu...............    6       1    Fm1
......................................................................ugagaucauugugaaaacugaGu...............    7       1    Fm1
......................................................................uNagaucauugugaaaacugauu...............    2       1    Fm1
......................................................................ugagaucauugugaaCacugauu...............    6       1    Fm1
......................................................................ugGgaucauugugaaaacugauu...............    82      1    Fm1
......................................................................ugagaucauugugaaaacugCuu...............    11      1    Fm1
......................................................................ugagaucauugugaaaacugauu...............    186320  0    Fm1
......................................................................ugagaucauugGgaaaacugauu...............    5       1    Fm1
......................................................................ugagaAcauugugaaaacugauu...............    20      1    Fm1
......................................................................ugagaucauugugaaaacAgauu...............    11      1    Fm1
......................................................................ugagaucCuugugaaaacugauu...............    5       1    Fm1
......................................................................ugagaucauAgugaaaacugauu...............    7       1    Fm1
......................................................................ugagaucauugugaaaacGgauu...............    12      1    Fm1
......................................................................Ngagaucauugugaaaacugauu...............    96      1    Fm1
......................................................................ugCgaucauugugaaaacugauu...............    26      1    Fm1
......................................................................ugagaGcauugugaaaacugauu...............    7       1    Fm1
......................................................................ugagaucauuguCaaaacugauu...............    7       1    Fm1
......................................................................ugagaucauugugaaaacugauC...............    66      1    Fm1
......................................................................ugagaucauugugaaGacugauu...............    24      1    Fm1
......................................................................ugagaNcauugugaaaacugauu...............    23      1    Fm1
......................................................................ugUgaucauugugaaaacugauu...............    32      1    Fm1
......................................................................ugagaucauuguAaaaacugauu...............    34      1    Fm1
......................................................................Agagaucauugugaaaacugauu...............    19      1    Fm1
......................................................................ugagaucauuCugaaaacugauu...............    162     1    Fm1
......................................................................ugagaucauugugaaaacugauuc..............    46      0    Fm1
......................................................................ugagaucauugugaaaacugauuU..............    15122   1    Fm1
......................................................................ugagaucauugugaaaacugauuG..............    88      1    Fm1
......................................................................ugagaucauugugaaaacugauuA..............    857     1    Fm1
.......................................................................Uagaucauugugaaaacu...................    1       1    Fm1
.......................................................................gagaucauugAgaaaacug..................    1       1    Fm1
.......................................................................Aagaucauugugaaaacug..................    1       1    Fm1
.......................................................................gagaucauuAugaaaacug..................    1       1    Fm1
.......................................................................gagaucauuguUaaaacug..................    1       1    Fm1
.......................................................................Uagaucauugugaaaacug..................    1       1    Fm1
.......................................................................gagaucauugugaaaacuU..................    1       1    Fm1
.......................................................................gagaucauugugaaaacug..................    65      0    Fm1
.......................................................................gagaucauugugaaaacuga.................    5       0    Fm1
.......................................................................gagaucauuUugaaaacuga.................    1       1    Fm1
.......................................................................gagaucauugugaaaacugau................    3       0    Fm1
.......................................................................Uagaucauugugaaaacugau................    1       1    Fm1
.......................................................................gagaucauugugaaaacugaA................    2       1    Fm1
```

Star Mature

```
uguuggcuguaaugaugaccuucagacgaaacugguuuucauaaugaccuuccagauuguugugcuauucugagaucauugugaaaacugauuccguugcccccaauc
.......................................................................gagaucauugugaaaacugauu...............   20     0    Fm1
.......................................................................Uagaucauugugaaaacugauu...............   4      1    Fm1
........................................................................agaucauugugaaaacug..................   1      0    Fm1
........................................................................Ugaucauugugaaaacug..................   1      1    Fm1
........................................................................Ugaucauugugaaaacuga.................   3      1    Fm1
........................................................................agaucauugugaaaacuga.................   2      0    Fm1
........................................................................Ugaucauugugaaaacugau................   2      1    Fm1
........................................................................Ugaucauugugaaaacugauu...............   3      1    Fm1
........................................................................agaucauugugaaaacugauu...............   8      0    Fm1
.........................................................................gaucauugugaaaacuga.................   2      0    Fm1

...............................cugguuuucauaaugacAuuccaga....................................................   1      1    Fm2
...............................cugguuuucauaaugaccuuccaga....................................................   33     0    Fm2
...................................uuuucauaaugaccuuccaga....................................................   10     0    Fm2
...................................................................uucugagaucauugugaaa......................   3      0    Fm2
...................................................................uucugagaucauugugaaaa.....................   5      0    Fm2
...................................................................uucAgagaucauugugaaaa.....................   1      1    Fm2
...................................................................uucugagaucauugugaaaac....................   5      0    Fm2
...................................................................uucugagaucauugugaUaacu..................   1      1    Fm2
...................................................................uucugagaucGuugugaaaacu..................   2      1    Fm2
...................................................................uuAugagaucauugugaaaacu..................   1      1    Fm2
...................................................................uucugagaucauugugaGaacu..................   2      1    Fm2
...................................................................uucugagaucauugugaCaacu..................   3      1    Fm2
...................................................................uucugGgaucauugugaaaacu..................   1      1    Fm2
...................................................................uuGugagaucauugugaaaacu..................   1      1    Fm2
...................................................................uucugagaucauugugCaaacu..................   6      1    Fm2
...................................................................uucugagaucauugugaaaacu..................   1133   0    Fm2
...................................................................uucugagaucaGugugaaaacu..................   1      1    Fm2
...................................................................uucAgagaucauugugaaaacu..................   1      1    Fm2
...................................................................uucugagaucauugugaaGacu..................   1      1    Fm2
...................................................................uucugagaucauugugGaaacu..................   1      1    Fm2
...................................................................uucugagaucUuugugaaaacu..................   1      1    Fm2
...................................................................uucugagaucauugugUaaacu..................   1      1    Fm2
...................................................................uucugagaucauugugaaaacG..................   8      1    Fm2
...................................................................uucugNgaucauugugaaaacu..................   1      1    Fm2
...................................................................uucuAagaucauugugaaaacu..................   2      1    Fm2
...................................................................uuAugagaucauugugaaaacug.................   1      1    Fm2
...................................................................uucugagaucauugugaaaacuA.................   2      1    Fm2
...................................................................uucugagaucauugugCaaacug.................   1      1    Fm2
...................................................................uucugagaucauugugaaaacug.................   98     0    Fm2
...................................................................uucugagaucauugugaaaacuU.................   19     1    Fm2
...................................................................uucugagaucauugugaGaacug.................   1      1    Fm2
.....................................................................cugagaucauugugaaaacug.................   12     0    Fm2
......................................................................ugagaucauugugaaaaU....................   5      1    Fm2
......................................................................ugagaucauugugaaaaA....................   81     1    Fm2
......................................................................ugagaucauugugaaaac....................   80     0    Fm2
......................................................................ugagaucauugugaaaaG....................   95     1    Fm2
......................................................................uUagaucauugugaaaacu...................   3      1    Fm2
......................................................................uCagaucauugugaaaacu...................   1      1    Fm2
......................................................................ugagaucauugugaaaUcu...................   11     1    Fm2
......................................................................ugagaucauugugaaaGcu...................   6      1    Fm2
......................................................................ugagaucauugCgaaaacu...................   1      1    Fm2
......................................................................ugagaucauugugaaaacA...................   1      1    Fm2
......................................................................ugagaAcauugugaaaacu...................   2      1    Fm2
......................................................................ugagaucauugugaaCacu...................   1      1    Fm2
......................................................................ugagauUauugugaaaacu...................   2      1    Fm2
......................................................................ugaAaucauugugaaaacu...................   1      1    Fm2
......................................................................ugagaucauugugaaUacu...................   1      1    Fm2
......................................................................ugagaucauugugaaaaUu...................   35     1    Fm2
......................................................................ugagaucauugAgaaaacu...................   1      1    Fm2
......................................................................ugagaucauugugaaaaAu...................   56     1    Fm2
......................................................................ugagaucauugugaaaCcu...................   9      1    Fm2
......................................................................ugagaNcauugugaaaacu...................   2      1    Fm2
......................................................................ugGgaucauugugaaaacu...................   1      1    Fm2
......................................................................ugagaucauugugCaaacu...................   2      1    Fm2
......................................................................ugagaucauugugaaaacG...................   57     1    Fm2
......................................................................ugagUucauugugaaaacu...................   1      1    Fm2
......................................................................uAagaucauugugaaaacu...................   1      1    Fm2
......................................................................ugagaucauugugaGaacu...................   1      1    Fm2
......................................................................ugagaucauuUugaaaacu...................   5      1    Fm2
```

Star **Mature**

uguuggcuguaaugaugaccuucagacgaaacugguuuucauaaugaccuuccagauuguugugcuauucugagaucauugugaaaacugauuccguugcccccaauc			
....ugagaucGuugugaaaacu...................	27	1	Fm2
....ugagCucauugugaaaacu...................	1	1	Fm2
....ugagaucauugugaaaacC...................	2	1	Fm2
....Agagaucauugugaaaacu...................	1	1	Fm2
....Ngagaucauugugaaaacu...................	2	1	Fm2
....ugagauGauugugaaaacu...................	1	1	Fm2
....ugUgaucauugugaaaacu...................	1	1	Fm2
....ugagaCcauugugaaaacu...................	1	1	Fm2
....ugagaucUuugugaaaacu...................	3	1	Fm2
....ugagaucauuguAaaaacu...................	1	1	Fm2
....Cgagaucauugugaaaacu...................	2	1	Fm2
....ugagaucauugugaaaaGu...................	30	1	Fm2
....ugagaucauugugaaaacu...................	5643	0	Fm2
....ugagGucauugugaaaacu...................	1	1	Fm2
....ugagaucauuCugaaaacu...................	7	1	Fm2
....ugagaucauCgugaaaacug..................	35	1	Fm2
....ugagUucauugugaaaacug..................	27	1	Fm2
....ugagauNauugugaaaacug..................	13	1	Fm2
....Cgagaucauugugaaaacug..................	60	1	Fm2
....ugagaucauugugaaaacug..................	211480	0	Fm2
....ugagaucauuCugaaaacug..................	196	1	Fm2
....ugagaucaAugugaaaacug..................	12	1	Fm2
....ugagaucauugugaaUacug..................	12	1	Fm2
....ugagaucauAgugaaaacug..................	16	1	Fm2
....Ngagaucauugugaaaacug..................	108	1	Fm2
....ugagauGauugugaaaacug..................	6	1	Fm2
....ugagaucauuguAaaaacug..................	25	1	Fm2
....ugagaucauugugaaaacuA..................	311	1	Fm2
....ugagauAauugugaaaacug..................	13	1	Fm2
....ugGgaucauugugaaaacug..................	46	1	Fm2
....ugagaucauugugCaaacug..................	9	1	Fm2
....ugagaucauugugaGaacug..................	37	1	Fm2
....ugUgaucauugugaaaacug..................	27	1	Fm2
....ugagaucauugGgaaaacug..................	9	1	Fm2
....uAagaucauugugaaaacug..................	86	1	Fm2
....ugagaucauugugaaaacCg..................	27	1	Fm2
....ugagaAcauugugaaaacug..................	15	1	Fm2
....ugagGucauugugaaaacug..................	37	1	Fm2
....ugagaucauugugaUaacug..................	9	1	Fm2
....ugagaucCuugugaaaacug..................	3	1	Fm2
....Agagaucauugugaaaacug..................	20	1	Fm2
....ugagaucauugugaaGacug..................	30	1	Fm2
....ugagaucauugugaaaUcug..................	380	1	Fm2
....ugaAaucauugugaaaacug..................	21	1	Fm2
....ugagauUauugugaaaacug..................	66	1	Fm2
....ugagaGcauugugaaaacug..................	5	1	Fm2
....Ggagaucauugugaaaacug..................	9	1	Fm2
....ugagaucauugugaaaacuU..................	533	1	Fm2
....ugaCaucauugugaaaacug..................	17	1	Fm2
....ugagaNcauugugaaaacug..................	32	1	Fm2
....ugagaucauugCgaaaacug..................	24	1	Fm2
....ugagaucUuugugaaaacug..................	23	1	Fm2
....ugagaucauGgugaaaacug..................	8	1	Fm2
....ugagCucauugugaaaacug..................	69	1	Fm2
....ugagaucauugugaaCacug..................	6	1	Fm2
....ugagaCcauugugaaaacug..................	26	1	Fm2
....ugagaucauugugaaaGcug..................	255	1	Fm2
....ugagaucauugugaaaacGg..................	961	1	Fm2
....uNagaucauugugaaaacug..................	1	1	Fm2
....ugagaucauugugaaaaUug..................	1035	1	Fm2
....ugagaucauuUugaaaacug..................	128	1	Fm2
....ugagaucaCugugaaaacug..................	18	1	Fm2
....ugaUaucauugugaaaacug..................	11	1	Fm2
....ugagaucauugugaaaacuC..................	34	1	Fm2
....ugagaucauugugaaaacAg..................	5	1	Fm2
....ugNgaucauugugaaaacug..................	2	1	Fm2
....ugagaucauugAgaaaacug..................	25	1	Fm2
....ugagaucaGugugaaaacug..................	19	1	Fm2
....uCagaucauugugaaaacug..................	18	1	Fm2
....ugagaucauugugGaaacug..................	28	1	Fm2

```
                                   Star                                Mature
uguuggcuguaaugaugaccuucagacgaaacugguuuucauaaaugaccuuccagauuguugugcuauucugagaucauugugaaaacugauuccguugcccccaauc
.......................................................................ugagaucauugugaaaaGug..................  994     1  Fm2
.......................................................................ugagaucauugugaaaCcug..................  369     1  Fm2
.......................................................................ugagaucauugugaCaacug..................  2       1  Fm2
.......................................................................ugagaucauuguCaaaacug..................  10      1  Fm2
.......................................................................ugCgaucauugugaaaacug..................  8       1  Fm2
.......................................................................uUagaucauugugaaaacug..................  49      1  Fm2
.......................................................................ugagaucauugugUaaacug..................  43      1  Fm2
.......................................................................ugagaucauugugaaaaAug..................  1706    1  Fm2
.......................................................................ugagaucauuAugaaaacug..................  388     1  Fm2
.......................................................................ugagaucGuugugaaaacug..................  41      1  Fm2
.......................................................................ugagaucauuguUaaaacug..................  16      1  Fm2
.......................................................................ugagaucCuugugaaaacuga.................  3       1  Fm2
.......................................................................ugagaucauugugaUaacuga.................  7       1  Fm2
.......................................................................Ngagaucauugugaaaacuga.................  47      1  Fm2
.......................................................................ugaCaucauugugaaaacuga.................  8       1  Fm2
.......................................................................ugagaucaAugugaaaacuga.................  9       1  Fm2
.......................................................................ugagaucauugugGaaacuga.................  15      1  Fm2
.......................................................................ugagaucauuguCaaaacuga.................  8       1  Fm2
.......................................................................ugUgaucauugugaaaacuga.................  13      1  Fm2
.......................................................................ugagaucGuugugaaaacuga.................  105     1  Fm2
.......................................................................ugagauGauugugaaaacuga.................  7       1  Fm2
.......................................................................ugagaucauugugaaaaUuga.................  529     1  Fm2
.......................................................................ugagaucauugCgaaaacuga.................  16      1  Fm2
.......................................................................ugagaucauugugaaaacAga.................  9       1  Fm2
.......................................................................ugagaucauugugaaaacCga.................  13      1  Fm2
.......................................................................ugagaucauuCugaaaacuga.................  97      1  Fm2
.......................................................................ugagaucaGugugaaaacuga.................  5       1  Fm2
.......................................................................ugagaCcauugugaaaacuga.................  7       1  Fm2
.......................................................................Cgagaucauugugaaaacuga.................  23      1  Fm2
.......................................................................ugagaucauugugaaaacuga.................  113491  0  Fm2
.......................................................................ugagaucauGgugaaaacuga.................  8       1  Fm2
.......................................................................ugagaucauugugCaaacuga.................  6       1  Fm2
.......................................................................ugaAaucauugugaaaacuga.................  24      1  Fm2
.......................................................................ugagaucauugugaaaacugU.................  1821    1  Fm2
.......................................................................uNagaucauugugaaaacuga.................  2       1  Fm2
.......................................................................ugagaucauugugaaaGcuga.................  129     1  Fm2
.......................................................................ugagaucauugugaaaacuUa.................  35      1  Fm2
.......................................................................ugagaucauugugaaaacuAa.................  14      1  Fm2
.......................................................................ugagaucauugugaCaacuga.................  1       1  Fm2
.......................................................................ugagaucauugugaGaacuga.................  18      1  Fm2
.......................................................................ugagUucauugugaaaacuga.................  26      1  Fm2
.......................................................................ugaUaucauugugaaaacuga.................  15      1  Fm2
.......................................................................ugagaucauuguAaaaacuga.................  14      1  Fm2
.......................................................................ugagaucauCgugaaaacuga.................  46      1  Fm2
.......................................................................ugagaucaCugugaaaacuga.................  19      1  Fm2
.......................................................................ugagaucauugugaaGacuga.................  19      1  Fm2
.......................................................................ugagaucauuAugaaaacuga.................  226     1  Fm2
.......................................................................ugagaucUuugugaaaacuga.................  21      1  Fm2
.......................................................................ugagaucauuUugaaaacuga.................  67      1  Fm2
.......................................................................Agagaucauugugaaaacuga.................  10      1  Fm2
.......................................................................uUagaucauugugaaaacuga.................  29      1  Fm2
.......................................................................ugagaNcauugugaaaacuga.................  16      1  Fm2
.......................................................................ugagCucauugugaaaacuga.................  38      1  Fm2
.......................................................................ugagaucauAgugaaaacuga.................  6       1  Fm2
.......................................................................ugagaucauugGgaaaacuga.................  9       1  Fm2
.......................................................................ugGgaucauugugaaaacuga.................  32      1  Fm2
.......................................................................ugagaucauugugaaaacGga.................  4       1  Fm2
.......................................................................ugagaAcauugugaaaacuga.................  9       1  Fm2
.......................................................................ugagaucauugugaaCacuga.................  2       1  Fm2
.......................................................................ugagaucauugugaaaacugG.................  5613    1  Fm2
.......................................................................ugagauNauugugaaaacuga.................  5       1  Fm2
.......................................................................ugagaucauugugUaaacuga.................  6       1  Fm2
.......................................................................ugagaucauugugaaaacugC.................  7       1  Fm2
.......................................................................ugagauAauugugaaaacuga.................  7       1  Fm2
.......................................................................ugagaucauugugaaaCcuga.................  192     1  Fm2
.......................................................................ugagGucauugugaaaacuga.................  16      1  Fm2
.......................................................................ugagaucauugugaaUacuga.................  8       1  Fm2
.......................................................................ugagaucauugugaaaaAuga.................  970     1  Fm2
.......................................................................ugagaucauugAgaaaacuga.................  8       1  Fm2
.......................................................................ugagaGcauugugaaaacuga.................  3       1  Fm2
```

Star **Mature**

```
uguuggcuguaaugaugaccuucagacgaaacugguuuucauaaugaccuuccagauuguugugcuauucugagaucauugugaaaacugauuccguugcccccaauc
......................................................................uCagaucauugugaaaacuga.................   8       1   Fm2
......................................................................ugagaucauugugaaaacuCa.................   2       1   Fm2
......................................................................uAagaucauugugaaaacuga.................   56      1   Fm2
......................................................................ugagaucauugugaaaUcuga.................   218     1   Fm2
......................................................................ugagaucauugugaaaNcuga.................   1       1   Fm2
......................................................................ugagaucauugugaaaaGuga.................   494     1   Fm2
......................................................................ugCgaucauugugaaaacuga.................   6       1   Fm2
......................................................................ugagauUauugugaaaacuga.................   29      1   Fm2
......................................................................ugNgaucauugugaaaacuga.................   1       1   Fm2
......................................................................ugagaucauuguUaaaacuga.................   9       1   Fm2
......................................................................ugagaucaAugugaaaacugau................   4       1   Fm2
......................................................................ugagaucauuguAaaaacugau................   9       1   Fm2
......................................................................uUagaucauugugaaaacugau................   20      1   Fm2
......................................................................ugagaucauugugaaaacuAau................   9       1   Fm2
......................................................................ugaAaucauugugaaaacugau................   19      1   Fm2
......................................................................uNagaucauugugaaaacugau................   1       1   Fm2
......................................................................ugagaucauugugaaaaUugau................   503     1   Fm2
......................................................................ugagaucauugugaaaacugaA................   585     1   Fm2
......................................................................ugagaucCuugugaaaacugau................   2       1   Fm2
......................................................................ugagaucaCugugaaaacugau................   9       1   Fm2
......................................................................ugagaucauGgugaaaacugau................   2       1   Fm2
......................................................................ugagaucauugugaaaacGgau................   1       1   Fm2
......................................................................uAagaucauugugaaaacugau................   36      1   Fm2
......................................................................ugagaucauugugaaaUcugau................   162     1   Fm2
......................................................................ugagaNcauugugaaaacugau................   16      1   Fm2
......................................................................ugagaucauugugaaaacugaG................   6       1   Fm2
......................................................................ugagaucauugugaaaacuCau................   6       1   Fm2
......................................................................ugagaucauuguCaaaacugau................   4       1   Fm2
......................................................................ugUgaucauugugaaaacugau................   12      1   Fm2
......................................................................ugagaucauCgugaaaacugau................   16      1   Fm2
......................................................................ugagaucauugugaaaCcugau................   187     1   Fm2
......................................................................ugCgaucauugugaaaacugau................   9       1   Fm2
......................................................................Cgagaucauugugaaaacugau................   43      1   Fm2
......................................................................ugagaGcauugugaaaacugau................   7       1   Fm2
......................................................................ugagaucauugugaaaacugCu................   1       1   Fm2
......................................................................ugagCucauugugaaaacugau................   30      1   Fm2
......................................................................ugagaucauugugaaUacugau................   16      1   Fm2
......................................................................ugGgaucauugugaaaacugau................   29      1   Fm2
......................................................................ugagaucauuCugaaaacugau................   93      1   Fm2
......................................................................ugagUucauugugaaaacugau................   14      1   Fm2
......................................................................ugagaucauugugaaaacugaC................   22      1   Fm2
......................................................................ugagaucauugugCaaacugau................   7       1   Fm2
......................................................................ugagaucauugAgaaaacugau................   15      1   Fm2
......................................................................ugagaucauuAugaaaacugau................   206     1   Fm2
......................................................................ugagauAauugugaaaacugau................   2       1   Fm2
......................................................................uCagaucauugugaaaacugau................   12      1   Fm2
......................................................................ugagaucGuugugaaaacugau................   50      1   Fm2
......................................................................ugagaucauugGgaaaacugau................   6       1   Fm2
......................................................................ugagaucauugugGaaacugau................   12      1   Fm2
......................................................................ugagaucauugugaaGacugau................   10      1   Fm2
......................................................................ugagaucauugugaaaacAgau................   3       1   Fm2
......................................................................ugagaucauugugaaaacugau................   101226  0   Fm2
......................................................................ugagauGauugugaaaacugau................   2       1   Fm2
......................................................................ugagGucauugugaaaacugau................   15      1   Fm2
......................................................................ugagaucauuUugaaaacugau................   72      1   Fm2
......................................................................ugagauUauugugaaaacugau................   29      1   Fm2
......................................................................ugagaucauugugUaaacugau................   16      1   Fm2
......................................................................ugagaucauuguUaaaacugau................   11      1   Fm2
......................................................................Agagaucauugugaaaacugau................   7       1   Fm2
......................................................................ugagaucauugugaUaacugau................   15      1   Fm2
......................................................................ugagaucauugugaaaacCgau................   7       1   Fm2
......................................................................ugagaucauugugaGaacugau................   9       1   Fm2
......................................................................ugagaucauugugaaaGcugau................   150     1   Fm2
......................................................................ugagaucauugCgaaaacugau................   21      1   Fm2
......................................................................ugNgaucauugugaaaacugau................   1       1   Fm2
......................................................................ugagaucauugugaaaacuUau................   26      1   Fm2
......................................................................ugagauNauugugaaaacugau................   2       1   Fm2
......................................................................ugagaucaGugugaaaacugau................   1       1   Fm2
......................................................................ugagaucauugugaCaacugau................   1       1   Fm2
......................................................................ugagaucUuugugaaaacugau................   20      1   Fm2
```

Star **Mature**

```
uguuggcuguaaugaugaccuucagacgaaacugguuuucauaaaugaccuuccagauuguugugcuauucugagaucauugugaaaacugauuccguugcccccaauc
.......................................................................ugaUaucauugugaaaacugau................	5	1	Fm2
.......................................................................Ngagaucauugugaaaacugau................	45	1	Fm2
.......................................................................ugagaucauAgugaaaacugau................	9	1	Fm2
.......................................................................ugagaCcauugugaaaacugau................	18	1	Fm2
.......................................................................ugagaucauugugaaaacugUu................	12	1	Fm2
.......................................................................ugagaucauugugaaaaGugau................	429	1	Fm2
.......................................................................ugagaucauugugaaaaAugau................	797	1	Fm2
.......................................................................ugagaAcauugugaaaacugau................	10	1	Fm2
.......................................................................ugagaucauugugaaaacugGu................	4	1	Fm2
.......................................................................ugaCaucauugugaaaacugau................	4	1	Fm2
.......................................................................ugagaucaCugugaaaacugauu...............	22	1	Fm2
.......................................................................ugagaucauugugaaaacugauC...............	21	1	Fm2
.......................................................................ugagaucauugugaaaacugCuu...............	5	1	Fm2
.......................................................................ugagaucauugugaaCacugauu...............	5	1	Fm2
.......................................................................ugUgaucauugugaaaacugauu...............	27	1	Fm2
.......................................................................ugagaucUuugugaaaacugauu...............	24	1	Fm2
.......................................................................ugagCucauugugaaaacugauu...............	51	1	Fm2
.......................................................................ugagaucauugGgaaaacugauu...............	4	1	Fm2
.......................................................................ugagaucauugugaaaacAgauu...............	6	1	Fm2
.......................................................................ugagaucauugugaaaacugaAu...............	31	1	Fm2
.......................................................................ugagaucaGugugaaaacugauu...............	9	1	Fm2
.......................................................................ugagauNauugugaaaacugauu...............	8	1	Fm2
.......................................................................ugagaucauugugaaaacugaGu...............	2	1	Fm2
.......................................................................Ggagaucauugugaaaacugauu...............	3	1	Fm2
.......................................................................ugagaucauugugUaaacugauu...............	38	1	Fm2
.......................................................................ugagaucauugAgaaaacugauu...............	9	1	Fm2
.......................................................................uUagaucauugugaaaacugauu...............	36	1	Fm2
.......................................................................ugaCaucauugugaaaacugauu...............	6	1	Fm2
.......................................................................ugagaucauugugaaaGcugauu...............	178	1	Fm2
.......................................................................ugagaucauugugaaaaGugauu...............	627	1	Fm2
.......................................................................ugagaucauCgugaaaacugauu...............	22	1	Fm2
.......................................................................Ngagaucauugugaaaacugauu...............	72	1	Fm2
.......................................................................ugagaucauugCgaaaacugauu...............	17	1	Fm2
.......................................................................ugagaAcauugugaaaacugauu...............	25	1	Fm2
.......................................................................ugCgaucauugugaaaacugauu...............	13	1	Fm2
.......................................................................ugagauAauugugaaaacugauu...............	6	1	Fm2
.......................................................................ugagaucauugugaaaacugauA...............	1569	1	Fm2
.......................................................................ugagaucauuAugaaaacugauu...............	290	1	Fm2
.......................................................................Cgagaucauugugaaaacugauu...............	32	1	Fm2
.......................................................................ugagaucauugugaaaUcugauu...............	319	1	Fm2
.......................................................................ugagaucCuugugaaaacugauu...............	2	1	Fm2
.......................................................................ugagUucauugugaaaacugauu...............	32	1	Fm2
.......................................................................ugagaucauugugaaaacuCauu...............	9	1	Fm2
.......................................................................ugagaucauugugaGaacugauu...............	27	1	Fm2
.......................................................................ugagaucauuUugaaaacugauu...............	100	1	Fm2
.......................................................................ugagaucauugugaaaNcugauu...............	1	1	Fm2
.......................................................................ugagaucauugugaaaacugauG...............	27	1	Fm2
.......................................................................ugagaucaAugugaaaacugauu...............	9	1	Fm2
.......................................................................Agagaucauugugaaaacugauu...............	16	1	Fm2
.......................................................................ugagaucauugugaaaacugGuu...............	7	1	Fm2
.......................................................................ugaAaucauugugaaaacugauu...............	19	1	Fm2
.......................................................................ugagaucGuugugaaaacugauu...............	281	1	Fm2
.......................................................................ugagaucauuguCaaacugauu...............	7	1	Fm2
.......................................................................ugagaucauugugaaaaAugauu...............	1217	1	Fm2
.......................................................................ugagaucauugugaUaacugauu...............	8	1	Fm2
.......................................................................ugagaucauugugCaaacugauu...............	9	1	Fm2
.......................................................................ugagaucauGgugaaaacugauu...............	4	1	Fm2
.......................................................................ugagaCcauugugaaaacugauu...............	10	1	Fm2
.......................................................................ugagauGauugugaaaacugauu...............	8	1	Fm2
.......................................................................ugagaucauuguUaaacugauu...............	12	1	Fm2
.......................................................................ugagaucauugugaaaacuAauu...............	7	1	Fm2
.......................................................................ugagaNcauugugaaaacugauu...............	23	1	Fm2
.......................................................................ugagaucauugugaCaacugauu...............	3	1	Fm2
.......................................................................ugagaucauugugGaaacugauu...............	23	1	Fm2
.......................................................................ugagaucauugugaaaacuUauu...............	27	1	Fm2
.......................................................................uAagaucauugugaaaacugauu...............	63	1	Fm2
.......................................................................ugagaucauuguAaaaacugauu...............	15	1	Fm2
.......................................................................ugagaucauugugaaaacGgauu...............	4	1	Fm2
.......................................................................ugagaucauugugaaaacCgauu...............	21	1	Fm2
.......................................................................ugagauUauugugaaaacugauu...............	98	1	Fm2
```

```
                                   Star                              Mature
uguuggcuguaaugaugaccuucagacgaaacugguuuucauaaugaccuuccagauuguugugcuauucugagaucauugugaaaacugauuccguugcccccaauc
......................................................................ugaUaucauugugaaaacugauu...............    7        1    Fm2
......................................................................ugGgaucauugugaaaacugauu...............    42       1    Fm2
......................................................................ugagaucauugugaaGacugauu...............    21       1    Fm2
......................................................................ugagGucauugugaaaacugauu...............    21       1    Fm2
......................................................................ugagaucauugugaaaacugUuu...............    14       1    Fm2
......................................................................uNagaucauugugaaaacugauu...............    1        1    Fm2
......................................................................ugagaucauuCugaaaacugauu...............    162      1    Fm2
......................................................................ugagaGcauugugaaaacugauu...............    6        1    Fm2
......................................................................ugagaucauugugaaaacugauu...............    143811   0    Fm2
......................................................................ugagaucauugugaaaaUugauu...............    705      1    Fm2
......................................................................ugagaucauAgugaaaacugauu...............    10       1    Fm2
......................................................................ugagaucauugugaaaacugaCu...............    16       1    Fm2
......................................................................ugagaucauugugaaaCcugauu...............    248      1    Fm2
......................................................................uCagaucauugugaaaacugauu...............    10       1    Fm2
......................................................................ugagaucauugugaaUacugauu...............    17       1    Fm2
......................................................................ugagaucauugugaaaacugauuA..............    790      1    Fm2
......................................................................ugagaucauugugaaaacugauuG..............    61       1    Fm2
......................................................................ugagaucauugugaaaacugauuc..............    59       0    Fm2
......................................................................ugagaucauugugaaaacugauuU..............    13895    1    Fm2
......................................................................ugagaucauugugaaaacugauucU.............    7        1    Fm2
.......................................................................gagaucauugugaaaaAug..................    1        1    Fm2
.......................................................................gagaucauugugaaaacuU..................    1        1    Fm2
.......................................................................gagaucauugCgaaaacug..................    1        1    Fm2
.......................................................................gagaucauugugaaaacGg..................    1        1    Fm2
.......................................................................gagaucauugugaaaacug..................    43       0    Fm2
.......................................................................Uagaucauugugaaaacug..................    7        1    Fm2
.......................................................................Uagaucauugugaaaacuga.................    2        1    Fm2
.......................................................................gagaucauugugaaaacuga.................    5        0    Fm2
.......................................................................Uagaucauugugaaaacugau................    5        1    Fm2
.......................................................................gagaucauugugaaaacugau................    3        0    Fm2
.......................................................................gagaucauugugCaaacugauu...............    1        1    Fm2
.......................................................................Uagaucauugugaaaacugauu...............    3        1    Fm2
........................................................................Ugaucauugugaaaacugau................    1        1    Fm2
........................................................................Ugaucauugugaaaacugauu...............    4        1    Fm2
........................................................................agaucauugugaaaacugauu...............    2        0    Fm2
```
